# Positive affect during adolescence and health and well-being in adulthood: An outcome-wide longitudinal approach

**DOI:** 10.1371/journal.pmed.1004365

**Published:** 2024-04-02

**Authors:** Eric S. Kim, Renae Wilkinson, Sakurako S. Okuzono, Ying Chen, Koichiro Shiba, Richard G. Cowden, Tyler J. VanderWeele

**Affiliations:** 1 Department of Psychology, University of British Columbia, Vancouver, British Columbia, Canada; 2 Human Flourishing Program, Institute for Quantitative Social Science, Harvard University, Cambridge, Massachusetts, United States of America; 3 Lee Kum Sheung Center for Health and Happiness, Harvard T.H. Chan School of Public Health, Boston, Massachusetts, United States of America; 4 Department of Social & Behavioral Sciences, Harvard T.H. Chan School of Public Health, Boston, Massachusetts, United States of America; 5 Department of Epidemiology, Harvard T.H. Chan School of Public Health, Boston, Massachusetts, United States of America; 6 Department of Epidemiology, Boston University School of Public Health, Boston, Massachusetts, United States of America; 7 Department of Biostatistics, Harvard T.H. Chan School of Public Health, Boston, Massachusetts, United States of America

## Abstract

**Background:**

Several intergovernmental organizations, including the World Health Organization and United Nations, are urging countries to use well-being indicators for policymaking. This trend, coupled with increasing recognition that positive affect is beneficial for health/well-being, opens new avenues for intervening on positive affect to improve outcomes. However, it remains unclear if positive affect in adolescence shapes health/well-being in adulthood. We examined if increases in positive affect during adolescence were associated with better health/well-being in adulthood across 41 outcomes.

**Methods and findings:**

We conducted a longitudinal cohort study using data from Add Health—a prospective and nationally representative cohort of community-dwelling U.S. adolescents. Using regression models, we evaluated if increases in positive affect over 1 year (between Wave I; 1994 to 1995 and Wave II; 1995 to 1996) were associated with better health/well-being 11.37 years later (in Wave IV; 2008; *N* = 11,040) or 20.64 years later (in Wave V; 2016 to 2018; *N* = 9,003). Participants were aged 15.28 years at study onset, and aged 28.17 or 37.20 years—during the final assessment. Participants with the highest (versus lowest) positive affect had better outcomes on 3 (of 13) physical health outcomes (e.g., higher cognition (β = 0·12, 95% CI = 0·05, 0·19, *p* = 0.002)), 3 (of 9) health behavior outcomes (e.g., lower physical inactivity (RR = 0·80, CI = 0·66, 0·98, *p* = 0.029)), 6 (of 7) mental health outcomes (e.g., lower anxiety (RR = 0·81, CI = 0·71, 0·93, p = 0.003)), 2 (of 3) psychological well-being (e.g., higher optimism (β *=* 0·20, 95% CI = 0·12, 0·28, *p* < 0.001)), 4 (of 7) social outcomes (e.g., lower loneliness (β *=* −0·09, 95% CI = −0·16, −0·02, *p* = 0.015)), and 1 (of 2) civic/prosocial outcomes (e.g., more voting (RR *=* 1·25, 95% CI = 1·16, 1·36, *p* < 0.001)). Study limitations include potential unmeasured confounding and reverse causality.

**Conclusions:**

Enhanced positive affect during adolescence is linked with a range of improved health/well-being outcomes in adulthood. These findings suggest the promise of testing scalable positive affect interventions and policies to more definitively assess their impact on outcomes.

## Abstract 2

Leveraging data from the U.S Add Health project, Eric Kim and colleagues explore how mood during adolescence influences health and well-being outcomes in adulthood.

## Author summary

**Why was this study done?:**

- Intergovernmental organizations, including the World Health Organization and United Nations, are advocating for the inclusion of well-being indicators in policy-making, alongside traditional economic measures like GDP.
- This trend, along with the growing understanding of positive affect’s benefits for health and well-being, creates exciting opportunities for intervening on positive affect to enhance outcomes, but, existing research primarily focuses on adult populations, and although some progress has been made, it remains unclear if positive affect assessed in adolescence shapes health/well-being in adulthood.
- Thus, there is a need to understand this knowledge gap to help inform relevant policies and interventions.

**What did the researchers do and find?:**

- In our longitudinal cohort study of U.S. adolescents from the Add Health project, we examined if increases in positive affect over 1 year during adolescence were associated with better health/well-being outcomes on 41 indicators in adulthood.
- In our analyses, we used data from 11,040 participants in Wave IV and 9,003 participants in Wave V, prioritizing the latest available data from Wave V whenever possible, and including Wave IV data when Wave V data was unavailable.
- The study, which spanned from adolescence (age 15.28) to adulthood (ages 28.17 or 37.20), found that higher positive affect is generally associated with better outcomes in various areas, including physical health, health behavior, mental health, psychological well-being, and social and civic engagement.

**What do these findings mean?:**

- Our findings indicate that fostering positive affect during adolescence could lead to a broad spectrum of improved health and well-being outcomes in adulthood, underscoring its potential value in youth-focused policies and interventions.
- The implications for practice include the potential development of targeted strategies to enhance positive affect among adolescents as a means to promote long-term health and well-being.
- However, limitations such as possible unmeasured confounding factors, possible reverse causality, and reliance on self-reported data suggest that these findings should be approached as preliminary, highlighting the need for further research in this area.

## Introduction

Three factors converge to highlight a unique opportunity for improving the health/well-being of adolescents and adults. First, several prominent intergovernmental organizations (e.g., Organization for Economic Co-operation and Development, World Health Organization, United Nations) are urging nations to use well-being indicators (e.g., happiness), in addition to traditional economic indicators (e.g., gross domestic product), when sculpting policies [1]. Many countries are adopting this paradigm shift. Second, according to the Lancet Commission on Adolescent Health and Well-being, “Failure to invest in the health of the largest generation of adolescents in the world’s history jeopardises earlier investments in maternal and child health, erodes future quality and length of life, and escalates suffering, inequality, and social instability” [2]. For this effort, identifying factors that foster health/well-being among adolescents is crucial. While much effort has focused on identifying risk factors of disease, investigators are increasingly seeking potentially modifiable health assets [3–7]. Targeting health assets during adolescence, a critical developmental phase for acquiring health assets and establishing healthy behaviors and mindsets, is a promising point of intervention that can enhance the trajectory of health/well-being across the life course. Third, positive affect—the experience of pleasurable emotions, such as happiness, joy, excitement, enthusiasm, calm, and contentment—is one promising health asset, and emerging research shows that it is associated with a range of health/well-being outcomes [8]. Although some progress has been made, it remains unclear if positive affect assessed in adolescence shapes a wide range of health/well-being outcomes in adulthood. Additionally, positive affect has been declining in younger populations over the past decade and this trend is an ever-growing concern [9]. Thus, as the number of governments focusing on well-being grows, so do opportunities to target positive affect for its own sake, as well as a means to enhance the health/well-being of adolescents and adults.

Positive affect is shaped by social structures and changing life circumstances [10], it is also modifiable through various interventions that can be applied among individuals (e.g., therapy, online exercises, physical activity) [11] and at a national scale (e.g., policies) [12]. Accumulating research conducted in adults indicates that high positive affect is linked to improved health behaviors (e.g., increased: medication adherence, physical activity, sleep, diet), enhanced biological function (e.g., healthier: immune function, inflammation levels, lipid levels), and decreased risk of chronic diseases (e.g., stroke, cardiovascular disease) and mortality [3,8,13]. A small number of landmark studies have prospectively evaluated how positive affect (and related constructs) in adolescents might influence subsequent health outcomes. For example, in adolescents, positive affect and psychological well-being composite scores (composed of elements such as positive affect, life satisfaction, purpose in life, optimism, etc.) have been linked with decreased risk of poor health outcomes (e.g., healthier cardiometabolic profiles, healthier BMI) [14–16]. However, evidence for associations with health behaviors are mixed. For example, positive affect has been linked with improved health behaviors (e.g., higher physical activity, healthier diets, not smoking, lower composite health behaviors index scores composed of factors like: physical inactivity, fast food consumption, binge drinking, smoking) [14,17,18], but there have also been interesting null results (e.g., no associations with: smoking, maintaining physical activity or becoming physically active, maintaining health diets, sleep duration) [14,19].

When considering prior studies conducted in younger populations, they have contributed substantially to the literature. However, they remain limited in some ways. First, many are cross-sectional, making it difficult to evaluate causality. Second, many health behavior and physical health outcomes have not yet been examined. Third, numerous studies do not account for key potential confounders (e.g., depressive symptoms/negative affect or baseline physical health). Fourth, many studies evaluated composite measures of psychological well-being (making it difficult to determine if specific aspects of psychological functioning are “active ingredients”) and/or outcomes (making it difficult to determine which specific outcomes are driving composite outcome scores). Fifth, we are unaware of longitudinal studies that analyzed data in a way that allows researchers to ask a different question of particular importance in this era of translational research: What health/well-being outcomes might we observe if positive affect were intervened upon?

To begin addressing this question, we used an outcome-wide analytic approach [20]. This is a hypothesis-generating, data-driven analytic approach aimed at discovering estimates of the outcomes we might expect to observe if positive affect was intervened upon. Promising findings can then undergo further investigation in future studies. We leveraged a large, prospective, and nationally representative sample of U.S. adolescents, and examined if increases in positive affect over 1 year during adolescence were associated with better subsequent health/well-being 11.37 years or 20.64 years later across 41 health/well-being outcomes.

## Methods

### Study population

We used data from Add Health, a prospective and nationally representative sample of U.S. adolescents in grades 7 to 12 during the 1994 to 1995 school year (Wave I), and this sample was followed into adulthood. Using a stratified random sampling approach, 80 high schools and 52 feeder middle schools were selected because they collectively capture a representative sample of U.S. adolescents in terms of ethnicity, urbanicity, school size, school type, and region of country. Among the 20,745 students who participated in Wave I in-home interviews, we excluded adolescents who did not: participate in the Wave II survey (in which the exposure variable positive affect was assessed, *n* = 6,009, year 1995 to 1996) or have valid survey weights at the respective outcome wave (Wave IV *n* = 3,698, year 2008; Wave V *n* = 5,733, year 2016 to 2018), resulting in a final analytic sample of *N* = 11,040 for Wave IV outcomes and *N* = 9,003 for Wave V outcomes (see Figs A and B in S1 Appendix for additional details). The reason we have 2 outcome waves is because we used data from the latest available wave, which was Wave V, whenever possible. However, 10 important outcomes were not assessed in Wave V, but assessed in Wave IV. In these cases, we used data from Wave IV.

Our study used data from 3 time points (t_0_, t_1_, and t_2_). Almost all covariates were assessed in the pre-baseline wave (t_0_, Wave I—when Add Health participants were in grades 7 to 12 and aged 11 to 21). However, 3 covariates, not assessed during Wave I, were retrospectively assessed in Wave IV. This choice was made because controlling for potential confounders in the pre-baseline wave establishes a clear temporal order between covariates, exposure, and outcomes that helps alleviate the risk of adjusting for a potential mediator [20]. The exposure, positive affect, was then assessed 1 year later in the baseline wave (t_1_, Wave II—when Add Health participants were adolescents and aged 12 to 21). All outcomes were assessed another 11.73 or 20.64 years later in the outcome waves (t_3_, Wave IV—when Add Health participants were early adults aged 24 to 32 years; t_3_, Wave V—when participants were early-midlife adults aged 33 to 43), depending on data availability. We recognize that defining adolescence, the phase of life that stretches between childhood and adulthood, has long posed a challenge. The definition of adolescence as 10 to 19 years of age originates from the mid-20th century, a period when patterns of adolescent growth and the timing of role transitions were starkly different than modern times. Thus, an expanded and more inclusive definition of adolescence with an upper limit of 21 (i.e., American Academy of Pediatrics and the US Department of Health and Human Services) [21] or even 24 (i.e., Lancet Commission on Adolescent Health and Wellbeing) [22] aligns more closely with contemporary patterns of adolescent biological growth and social role transitions. Additionally, only 1.1% of our study sample (*n* = 118) was outside the ages of 10 to 19 at study baseline.

Add Health provides extensive documentation about their protocol, instrumentation, and complex sampling strategy elsewhere (https://addhealth.cpc.unc.edu/). Add Health has been approved by several ethics committees, including the University of North Carolina IRB. Further, informed consent was obtained from all respondents.

### Measures

#### Positive affect

The exposure was assessed at baseline (t_1_; Wave II) and also at pre-baseline (t_0_; Wave I) using the positive affect subscale of the Center for Epidemiological Studies Depression (CES-D) Scale—[23] a subscale that repeatedly emerges in factor analytic studies as illustrated in meta-analyses [24] and has been used repeatedly in past research [25]. Using a four-point scale (range 1 to 4), respondents rated the degree to which they experienced the following items in the past week: “I was happy,” “I felt hopeful about the future,” “I felt that I was just as good as other people,” “I enjoyed life.” We averaged all responses and created a composite score so that higher scores reflect higher positive affect (range 1 to 4). The Cronbach’s α coefficient, which assesses the internal consistency reliability of the scale, was 0.73. To examine potential threshold effects, we created tertiles based on the distribution of positive affect scores in the sample.

#### Covariates

Covariates were assessed in the pre-baseline wave (t_0_; Wave I, the closest wave before the exposure assessment), unless otherwise noted, and included: sociodemographic and family factors (age, sex, race/ethnicity (White, Black, Hispanic, Asian, Other), born in the U.S., geographic region (Northeast, Midwest, South, West), two-parent household, number of siblings, household income quintile, household welfare receipt, health insurance, smoker in household, mother age, mother race/ethnicity, parents born in the U.S., parental education (<high school, high school, some college, ≥college), mother employed full time, mother religious service attendance (never or seldom, <1×/week, ≥1×/week), mother self-rated health, mother happiness, parent has a disability, parent has obesity, parent has alcoholism, childhood maltreatment by parents (assessed retrospectively at Wave IV), psychosocial and academic factors (including mental health condition diagnosis [assessed retrospectively at Wave IV], negative affect, self-esteem, life expectancy, parental control, relationship quality with parent, religious service attendance, has romantic partner, has a learning disability, cognitive development [assessed with Peabody Picture Vocabulary Test [PPVT]], school connectedness, GPA, delinquency), and health status and health behaviors (somatic symptoms, pubertal development range, physical health condition diagnosis [assessed retrospectively at Wave IV], overweight/obesity, functional limitations, self-rated health, suicidal ideation, sleep disturbance, physical inactivity, cigarette smoking, binge drinking, marijuana use, illicit drug use, history of STIs, preventative health care use).

#### Outcomes

We considered 41 outcomes which were assessed in the outcome waves (t_2_; Wave IV or Wave V), and include dimensions of: physical health (number of diagnosed physical health conditions, cancer, high cholesterol, hypertension, diabetes, asthma, sleep apnea, migraines, allostatic load, overweight/obesity, functional limitations, cognition, self-rated health), health behavior (sleep disturbance, physical inactivity, cigarette smoking, binge drinking, marijuana use, prescription drug misuse, illicit drug use, history of sexually transmitted infections [STIs], preventative health care use), mental health (depression diagnosis, anxiety diagnosis, posttraumatic stress disorder [PTSD] diagnosis, attention deficit hyperactivity disorder [ADD/ADHD] diagnosis, negative affect, suicidal ideation, perceived stress), psychological well-being (optimism, job satisfaction, sense of control), social factors (relationship quality with parent, social activities, social support, loneliness, romantic relationship quality, satisfaction with parenting, perceived discrimination), and civic and prosocial behavior (voting, volunteering). A full description of each outcome can be found in Text A in S1 Appendix. These outcomes were chosen because they are frequently included in the conceptualization of key models that characterize the antecedents, processes, and outcomes that foster positive adolescent and adult development [26–28].

### Statistical analysis

We used an outcome-wide analytic approach [20], which has several characteristics not widely used outside of biostatistics and causal inference. Thus, we summarize those characteristics here. First, we run a separate model for each outcome and consistently use the same set of covariates across all models, and all outcomes. Second, we control for covariates in the wave prior to the exposure since, if we assess potential confounders in the same time point as the exposure (t_1_), it remains unclear if they are confounders or mediators; if we accidentally control for mediators in the same time point, we may spuriously attenuate true effects. A pragmatic approach to avoiding this problem is by adjusting for potential confounders in the pre-baseline wave (t_0_). Third, to enhance our ability to strive toward “no unmeasured confounding,” and “exchangeability” (as well as other criteria described in “disjunctive cause criterion” for selection of covariates that includes potential causes of either the exposure or the outcomes or both), which all enhance our ability to make causal inference, we adjust for a sufficiently rich set of potential confounder variables to make these assumptions plausible [29,30]. Fourth, to reduce potential reverse causality we also adjust for all outcome variables in the pre-baseline wave (t_0_). Fifth, to evaluate potential “change” in positive affect we adjust for positive affect in the pre-baseline wave (t_0_). This helps “hold constant” pre-baseline levels of positive affect (see Text B in S1 Appendix for proof). Adjusting for pre-baseline levels of positive affect (t_0_) also has several other advantages including helping reduce risk of reverse causality and also “removing” the accumulating effects positive affect already had on outcomes in the past (“prevalent exposure”) and allowing readers to instead focus on the effects of change in positive affect (“incident exposure”) over 1 year, on outcomes.

Separate models were run for each outcome. Depending on the nature of the outcome, a different model was run: (1) for each binary outcome with a prevalence <10%, logistic regression was used; (2) for each binary outcome with a prevalence ≥10%, generalized linear model (with a log link and Poisson distribution) was used; and (3) for each continuous outcome, a linear regression model was used. Further, each continuous outcome was standardized (mean = 0, SD = 1). All analyses were weighted to account for unequal probability of selection and attrition and adjust for the complex sampling design.

In our results section, we comment on the traditional 0.05 *p*-value threshold and provide 95% confidence intervals for all effect estimates, which are often considered preferable assessments of uncertainty since all thresholds are ultimately arbitrary. The study is reported according to the Strengthening the Reporting of Observational Studies in Epidemiology (STROBE) guideline (Checklist A in S1 Appendix). This study did not have a formally registered prospective protocol or analysis plan. However, all analyses were discussed and planned before we began analyses.

#### Secondary analyses

We carried out several additional analyses. First, we performed sensitivity analysis using *E*-values to evaluate the exposure–outcome association’s robustness to unmeasured confounding [31]. Second, we reanalyzed all models using only complete cases to evaluate the potential impact of using multiple imputation for handling missing data. Third, to provide a baseline comparison, we reanalyzed all models without any control for potential confounders. Fourth, we reanalyzed all models using a reduced list of potential confounders more conventionally used in the social/behavioral sciences (i.e., sociodemographic factors) to evaluate how similar (or different) our results were to past research. Fifth, we calculated the Number Needed to Treat (NNT) and Number Needed to Harm (NNH) for each outcome.

#### Multiple imputation

In our dataset, the percentage of missing data varied across variables and ranged from 0% to 26% (see Table A in S1 Appendix). We imputed missing data for the covariates and outcomes using an imputation by chained equations procedure by generating 5 datasets. This method provides a more flexible approach than other methods of handling missing data [32]. All analyses were conducted in Stata 17.

## Results

In our study, the final cohort consisted of 11,040 participants for Wave IV outcomes and 9,003 participants for Wave V outcomes. At the pre-baseline wave, when the potential confounders were assessed, participants were 15 years old (SD = 1·60), and just over half were women (*n* = 5,901; 53.45%). Participants reported being White (*n* = 5,960; 54.02%), Black (*n* = 2,253; 20.42%), Hispanic (*n* = 1,745; 15.82%), Asian (*n* = 675; 6.12%), and “Other” (*n* = 400; 3.63%). The distribution of sociodemographic and health characteristics at pre-baseline was generally consistent across positive affect tertiles, but there were some key differences: those in the highest (versus lowest) positive affect tertile had higher household income (e.g., 23% versus 14% were in the highest income quintile) and had a higher percentage of two-parent households (75% versus 64%). Table 1 describes the distribution of covariates. Table B in S1 Appendix shows how positive affect changed from the pre-baseline to baseline wave. The average length of follow-up from baseline (Wave II) to the outcome waves was 11.37 years (for Wave IV outcomes; SD = 0.50, range 10 to 12) or 20.64 years (for Wave V outcomes; SD = 0.71, range = 19 to 22).

**Table 1:** Characteristics of participants at pre-baseline by tertiles of baseline positive affect (National Longitudinal Study of Adolescent to Adult Health [Add Health]).

	Positive affect			
Characteristic	Tertile 1 (*n* = 4,184)	Tertile 2 (*n* = 4,274)	Tertile 3 (*n* = 2,575)	*p*-value
**Sociodemographic and family factors**				
Age (range: 11–21), median (Q1–Q3)	15 (14, 17)	15 (14, 16)	15 (14, 16)	<0.001
Female, *n* (%)	2,535 (56.24)	2,179 (50.98)	1,366 (53.05)	<0.001
Race/ethnicity, *n* (%)				<0.001
White	2,011 (48.10)	2,397 (56.12)	1,551 (60.26)	
Black	852 (20.38)	839 (19.64)	559 (21.72)	
Hispanic	826 (19.76)	654 (15.31)	264 (10.26)	
Asian	342 (8.18)	225 (5.27)	107 (4.16)	
Other	150 (3.59)	156 (3.65)	93 (3.61)	
Born in the U.S., *n* (%)	3,799 (90.84)	4,003 (93.68)	2,435 (94.56)	<0.001
Geographic region, *n* (%)				<0.001
Northeast	1,071 (25.60)	1,008 (23.58)	466 (18.10)	
Midwest	995 (23.78)	1,140 (26.67)	765 (29.71)	
South	1,563 (37.36)	1,567 (36.66)	1,011 (39.26)	
West	555 (13.26)	559 (13.08)	333 (12.93)	
Two-parent household, *n* (%)	2,700 (64.58)	2,969 (69.50)	1,865 (72.48)	<0.001
Number of siblings (range: 0–12), median (Q1–Q3)	1 (1,2)	1 (1,2)	1 (1,2)	<0.001
Household income, *n* (%)				<0.001
1st quintile	783 (24.88)	592 (17.46)	307 (14.87)	
2nd quintile	701 (22.28)	670 (19.76)	354 (17.15)	
3rd quintile	638 (20.27)	715 (21.09)	422 (20.45)	
4th quintile	520 (16.52)	670 (19.76)	457 (22.14)	
5th quintile	505 (16.05)	743 (21.92)	524 (25.39)	
Household welfare receipt, *n* (%)	956 (27.07)	731 (19.27)	386 (16.86)	<0.001
Has health insurance, *n* (%)	3,131 (86.02)	3,436 (89.04)	2,127 (90.82)	<0.001
Smoker in household, *n* (%)	1,745 (48.54)	1,679 (43.77)	952 (41.02)	<0.001
Mother age (range: 23–81), median (Q1–Q3)	40 (37,45)	41 (37,45)	41 (38,45)	0.005
Mother race/ethnicity, *n* (%)				<0.001
White	1,757 (54.60)	2,215 (63.03)	1,418 (66.35)	
Black	596 (18.52)	611 (17.39)	417 (19.51)	
Hispanic	604 (18.77)	454 (12.92)	188 (8.80)	
Asian	188 (5.84)	152 (4.33)	61 (2.85)	
Other	73 (2.27)	82 (2.33)	53 (2.48)	
Parents born in the U.S., *n* (%)	2,869 (72.47)	3,329 (80.57)	2,097 (83.98)	<0.001
Parental education, *n* (%)				<0.001
Less than high school	627 (15.11)	411 (9.67)	156 (6.07)	
High school equivalency	1,261 (30.39)	1,071 (25.19)	534 (20.76)	
Some college	1,038 (25.01)	1,147 (26.98)	686 (26.67)	
College degree or higher	1,224 (29.49)	1,623 (38.17)	1,196 (46.50)	
Mother employed full-time, *n* (%)	2,336 (56.28)	2,492 (58.65)	1,547 (60.52)	0.002
Mother religious service attendance, *n* (%)				0.001
Never or seldom	653 (20.09)	641 (18.17)	353 (16.41)	
Less than once a week	1,393 (42.86)	1,516 (42.98)	894 (41.56)	
At least once a week	1,204 (37.05)	1,370 (38.84)	904 (42.03)	
Mother self-rated health (range: 1–5), median (Q1–Q3)	3 (3, 4)	4 (3, 4)	4 (3, 5)	<0.001
Mother happy, *n* (%)	3,054 (85.52)	3,396 (88.55)	2,104 (91.08)	<0.001
Parent has a disability, *n* (%)	631 (15.88)	491 (11.87)	306 (12.26)	<0.001
Parent has obesity, *n* (%)	850 (24.43)	859 (23.22)	539 (23.92)	0.482
Parent has alcoholism, *n* (%)	582 (17.59)	599 (16.84)	301 (13.92)	0.001
Childhood maltreatment by parents, *n* (%)	979 (23.77)	836 (19.76)	445 (17.43)	<0.001
**Psychosocial and academic factors**				
Mental health condition diagnosis^a^^,^^b^, *n* (%)	251 (6.00)	197 (4.61)	109 (4.23)	0.001
Negative affect^b^ (range: 0–3), median (Q1–Q3)	1.71 (1.29, 2.00)	1.43 (1.14, 1.71)	1.29 (1.14, 1.57)	<0.001
Self-esteem (range: 1–5), median (Q1–Q3)	4 (3.50, 4.33)	4.17 (3.83, 4.50)	4.5 (4.00, 4.83)	<0.001
Life expectancy (range: 1–5), median (Q1–Q3)	4.5 (4.00, 5.00)	4.50 (4.00, 5.00)	4.50 (4.50, 5.00)	<0.001
Parental control (range: 0–7), median (Q1–Q3)	2 (1, 3)	2 (1, 3)	2 (1, 3)	<0.001
Neighborhood social cohesion (range: 0–5), median (Q1–Q3)	4 (3, 5)	4 (3, 5)	4 (3, 5)	<0.001
Relationship quality with a parent^a^ (range: 1–5), median (Q1–Q3)	5 (4, 5)	5 (4, 5)	5 (5, 5)	<0.001
Religious service attendance, *n* (%)				<0.001
Never or seldom	1,132 (27.09)	987 (23.14)	503 (19.54)	
Less than once a week	1,510 (36.13)	1,596 (37.41)	901 (35.00)	
At least once a week	1,537 (36.78)	1,683 (39.45)	1,170 (45.45)	
Has romantic partner, *n* (%)	1,441 (34.82)	1,478 (34.89)	844 (33.15)	0.283
Has a learning disability, *n* (%)	564 (15.72)	415 (10.87)	185 (7.97)	<0.001
PPVT (range: 13–146), median (Q1–Q3)	96 (87,108)	102 (93,112)	105 (96,115)	<0.001
School connectedness (range: 1–5), median (Q1–Q3)	3.60 (3.00, 4.00)	3.80 (3.40, 4.20)	4.00 (3.60, 4.40)	<0.001
GPA (range: 1–4), median (Q1–Q3)	2.67 (2.00, 3.25)	2.75 (2.25, 3.50)	3.00 (2.50, 3.67)	<0.001
Delinquency (range: 0–15), median (Q1–Q3)	3 (1, 5)	2 (1, 4)	1 (0, 3)	<0.001
**Health status and health behavior**				
Somatic symptoms (range: 0–4), median (Q1–Q3)	1.82 (1.55, 2.09)	1.73 (1.45, 2)	1.64 (1.45, 1.91)	<0.001
Pubertal development (range: −10.23–9.59), median (Q1–Q3)	−0.03 (−1.54, 1.29)	0.23 (−1.14, 1.42)	0.36 (−0.83, 1.68)	<0.001
Physical health condition diagnosis^a^^,^^b^, *n* (%)	822 (23.12)	867 (22.88)	512 (22.16)	0.686
Overweight/obesity^b^, *n* (%)	940 (23.30)	911 (21.82)	479 (18.94)	<0.001
Functional limitations^b^, *n* (%)	33 (0.80)	19 (0.45)	13 (0.51)	0.091
Self-rated health^b^ (range: 1–5), median (Q1–Q3)	4 (3, 4)	4 (3, 5)	4 (4, 5)	<0.001
Suicidal ideation^b^, *n* (%)	800 (19.32)	485 (11.43)	201 (7.85)	<0.001
Sleep disturbance^b^, *n* (%)	1,258 (30.08)	988 (23.12)	452 (17.56)	<0.001
Physical inactivity^b^, *n* (%)	259 (6.19)	189 (4.42)	96 (3.73)	<0.001
Cigarette smoking^b^, *n* (%)	802 (19.33)	632 (14.87)	281 (10.97)	<0.001
Binge drinking^b^, *n* (%)	294 (7.04)	223 (5.23)	103 (4.00)	<0.001
Marijuana use^b^, *n* (%)	751 (17.95)	580 (13.57)	266 (10.33)	<0.001
Illicit drug use^b^, *n* (%)	546 (13.32)	482 (11.43)	186 (7.31)	<0.001
History of STIs^a^, *n* (%)	116 (2.78)	89 (2.08)	41 (1.59)	0.004
Preventative health care use^a^, *n* (%)	2,561 (61.43)	2,850 (66.79)	1,820 (70.82)	<0.001

Table is based on non-imputed data. Range for each tertile of positive affect: tertile 1: 1.00–2.75; tertile 2: 3.00–3.50; tertile 3: 3.67–4.00. The statistics presented are unweighted and sample is restricted to participants surveyed at the exposure wave (Wave II) and first outcome wave (Wave IV), and who had complete data on the exposure (*N* = 11,033). The difference in sample size between Table 1 and the outcomes from Table 2 that apply the same sample restrictions (i.e., participation in Wave II and Wave IV) relates to the 7 participants who had missing data on the exposure measure and had those data imputed for the main analysis, resulting in an overall sample size for Wave IV outcomes of *N* = 11,040. No proportion varied by more than +/− 4% when restricting the sample by Wave V participation instead of Wave IV participation. See Table A in S1 Appendix for missing data description, and *p-*values in this table come from χ2 or analysis of variance tests. Cumulative percentages for categorical variables may not add up to 100% due to rounding.

^a^Pre-baseline covariate for Wave IV outcome.

^b^Pre-baseline covariate for Wave V outcome.

PPVT, Peabody Picture Vocabulary Test; STI, sexually transmitted infection.

Table 2 shows the associations between positive affect and subsequent health/well-being outcomes. When considering physical health outcomes and health behaviors, positive affect was associated with healthier functioning, or score, on 3 (out of 13) physical health outcomes, including lower likelihood of migraines (relative risk [*RR*] *=* 0·79, 95% confidence interval [CI] = 0·67, 0·93, *p* = 0.005), as well has higher cognition (*β =* 0·12, 95% CI = 0·05, 0·19, *p* = 0.002), and self-rated health (*β =* 0·11, 95% CI = 0·05, 0·18, *p* < 0.001). However, there was little evidence of an association with number of diagnosed physical health conditions, any of the other specific physical health conditions (i.e., cancer, high cholesterol, hypertension, diabetes, asthma, and sleep apnea), allostatic load, overweight/obesity, or functional limitations. When considering health behaviors, positive affect was associated with healthier functioning, or scores, on 3 (out of 9) health behavior outcomes, including: lower likelihood of prescription drug misuse (*RR =* 0·72, 95% CI = 0·56, 0·93, *p* = 0.013), lower likelihood of physical inactivity (*RR =* 0·80, 95% CI = 0·66, 0·98, *p* = 0.029), and lower likelihood of sleep disturbance (*RR =* 0·91, 95% CI = 0·85, 0·97, *p* = 0.004). However, there was little evidence of an association with cigarette smoking, binge drinking, marijuana use, illicit drug use, history of STIs, and preventative healthcare use.

**Table 2:** Associations of positive affect in adolescence with subsequent health and well-being in adulthood (National Longitudinal Study of Adolescent to Adult Health [Add Health]).

	Positive affect						
	Tertile 1^a^	Tertile 2^b^			Tertile 3^c^		
Outcome	(Reference)	β [95% CI]	RR/OR [95% CI]	*p*-value	β [95% CI]	RR/OR [95% CI]	*p*-value
**Physical health**							
Number of diagnosed physical health conditions	0.00	−0.03 [−0.09, 0.04]	-	0.431	−0.04 [−0.11, 0.03]	-	0.239
Cancer	1.00	-	1.24 [0.75, 2.03]	0.403	-	1.02 [0.58, 1.77]	0.957
High cholesterol	1.00	-	0.94 [0.75, 1.18]	0.610	-	0.91 [0.72, 1.15]	0.438
Hypertension	1.00	-	0.89 [0.77, 1.02]	0.087	-	0.90 [0.79, 1.02]	0.102
Diabetes	1.00	-	1.24 [0.93, 1.66]	0.134	-	1.16 [0.79, 1.70]	0.457
Asthma	1.00	-	0.99 [0.85, 1.15]	0.859	-	0.95 [0.79, 1.14]	0.558
Sleep apnea	1.00	-	1.02 [0.85, 1.22]	0.813	-	0.97 [0.78, 1.21]	0.767
Migraines^d^	1.00	-	0.84 [0.72, 0.99]	0.034	-	0.79 [0.67, 0.93]	0.005
Allostatic load	0.00	−0.04 [−0.12, 0.04]	-	0.266	−0.06 [−0.14, 0.02]	-	0.149
Overweight/obesity	1.00	-	0.99 [0.91, 1.09]	0.864	-	0.99 [0.91, 1.07]	0.802
Functional limitations	1.00	-	1.08 [0.90, 1.30]	0.412	-	0.84 [0.69, 1.01]	0.066
Cognition^d^	0.00	0.09 [−0.01, 0.17]	-	0.019	0.12 [0.05, 0.19]	-	0.002
Self-rated health	0.00	−0.00 [−0.06, 0.06]	-	0.916	0.11 [0.05, 0.18]	-	<0.001
**Health behavior**							
Sleep disturbance	1.00	-	0.96 [0.90, 1.02]	0.172	-	0.91 [0.85, 0.97]	0.004
Physical inactivity	1.00	-	0.82 [0.68, 1.00]	0.005	-	0.80 [0.66, 0.98]	0.029
Cigarette smoking	1.00	-	0.92 [0.83, 1.03]	0.141	-	0.93 [0.83, 1.05]	0.258
Binge drinking	1.00	-	0.74 [0.61, 0.91]	0.005	-	0.85 [0.68, 1.05]	0.123
Marijuana use	1.00	-	1.00 [0.88, 1.13]	0.966	-	1.00 [0.87, 1.15]	0.962
Prescription drug misuse	1.00	-	0.93 [0.76, 1.14]	0.481	-	0.72 [0.56, 0.93]	0.013
Illicit drug use	1.00	-	0.92 [0.64, 1.32]	0.638	-	0.92 [0.61, 1.38]	0.674
History of STIs^d^	1.00	-	1.15 [0.97, 1.36]	0.104	-	1.10 [0.92, 1.31]	0.307
Preventative health care use^d^	1.00	-	0.98 [0.93, 1.03]	0.448	-	0.96 [0.92, 1.01]	0.158
**Mental health**							
Depression diagnosis	1.00	-	0.98 [0.87, 1.10]	0.737	-	0.84 [0.74, 0.94]	0.004
Anxiety diagnosis	1.00	-	0.88 [0.78, 0.99]	0.038	-	0.81 [0.71, 0.93]	0.003
PTSD diagnosis	1.00	-	0.86 [0.65, 1.15]	0.313	-	0.63 [0.46, 0.85]	0.003
ADD/ADHD diagnosis^d^	1.00	-	0.87 [0.60, 1.27]	0.472	-	0.66 [0.45, 0.98]	0.039
Negative affect	0.00	−0.05 [−0.12, 0.01]	-	0.113	−0.17 [−0.24, −0.10]	-	<0.001
Suicidal ideation	1.00	-	0.74 [0.54, 1.00]	0.052	-	0.74 [0.52, 1.05]	0.089
Perceived stress	0.00	−0.12 [−0.18, −0.05]	-	<0.001	−0.23 [−0.30, −0.16]	-	<0.001
**Psychological well-being**							
Optimism	0.00	0.07 [−0.00, 0.14]	-	0.060	0.20 [0.12, 0.28]	-	<0.001
Job satisfaction^d^	0.00	0.08 [0.02, 0.14]	-	0.015	0.06 [−0.01, 0.13]	-	0.081
Sense of control^d^	0.00	0.08 [0.02, 0.13]	-	0.009	0.18 [0.13, 0.24]	-	<0.001
**Social factors**							
Relationship quality with parent	0.00	0.06 [−0.02, 0.14]	-	0.168	0.04 [−0.03, 0.12]	-	0.279
Social activities	1.00	-	1.00 [0.92, 1.08]	0.973	-	1.06 [0.98, 1.14]	0.142
Social support	0.00	0.06 [0.01, 0.11]	-	0.024	0.08 [0.02, 0.13]	-	0.008
Loneliness^d^	0.00	−0.02 [−0.08, 0.05]	-	0.575	−0.09 [−0.16, −0.02]	-	0.015
Romantic relationship quality^d^	0.00	0.05 [−0.01, 0.11]	-	0.109	0.05 [−0.01, 0.12]	-	0.121
Satisfaction with parenting^d^^,^^e^	0.00	0.06 [−0.04, 0.16]	-	0.241	0.16 [0.08, 0.25]	-	<0.001
Perceived discrimination	0.00	−0.04 [−0.11, 0.03]	-	0.251	−0.10 [−0.18, −0.02]	-	0.016
**Civic and prosocial behavior**							
Voting	1.00	-	1.12 [1.04, 1.21]	0.005	-	1.25 [1.16, 1.36]	<0.001
Volunteering	1.00	-	1.21 [1.10, 1.33]	<0.001	-	1.06 [0.96, 1.18]	0.260

Outcomes were derived from Wave V and models were weighted by the Wave V sample weight unless otherwise noted. Outcomes associated with Wave V: *N* = 9,003; outcomes associated with Wave IV: *N* = 11,040.

The analytic sample was restricted to those who participated in the survey at the exposure wave (Wave II) and had a valid sampling weight at the outcome wave from which the data for the respective outcome was derived (Wave IV or Wave V). Multiple imputation was performed to impute missing data on the covariates, exposure, and outcomes. All models controlled for sociodemographic and family factors (age, sex, race/ethnicity, nativity status, geographic region, family structure, number of siblings, household income, household welfare receipt, insurance status, smoker in household, mother age, mother race/ethnicity, parent nativity, parental education, mother employment status, mother religious service attendance, mother health status, mother happiness, parent has a disability, parent has obesity, parent has alcoholism, childhood maltreatment by parents), psychosocial and academic factors (mental health condition diagnosis, negative affect, self-esteem, life orientation, relationship quality with a parent, parental control, neighborhood social cohesion, religious service attendance, romantic relationship status, has a learning disability, PPVT, school connectedness, GPA, delinquency), health status and health behavior (somatic symptoms, pubertal development, physical health condition diagnosis, overweight/obesity, functional limitations, self-rated health, suicidal ideation, sleep disturbance, physical inactivity, cigarette smoking, binge drinking, marijuana use, illicit drug use, history of STIs, preventative health care use), and positive affect assessed at Wave I.

An outcome-wide analytic approach was used, and a separate model was run for each outcome. A different type of model was run depending on the nature of the outcome: (1) for each binary outcome with a prevalence of ≥10%, a generalized linear model (with a log link and Poisson distribution) was used to estimate an RR; (2) for each binary outcome with a prevalence of <10%, a logistic regression model was used to estimate an OR; and (3) for each continuous outcome, a linear regression model was used to estimate a β.

All continuous outcomes were standardized (mean = 0, standard deviation = 1), and β was the standardized effect size.

^a^For outcomes associated with Wave V: Tertile 1 *n* = 3,354; for outcomes associated with Wave IV: Tertile 1 *n* = 4,186.

^b^For outcomes associated with Wave V: Tertile 2 *n* = 2,422; for outcomes associated with Wave IV: Tertile 2 *n* = 3,015.

^c^For outcomes associated with Wave V: Tertile 3 *n* = 3,227; for outcomes associated with Wave IV: Tertile 3 *n* = 3,839.

^d^Outcome was derived from data from Wave IV and model was weighted by the Wave IV sample weight because the data for this outcome was not collected at Wave V.

^e^Analysis for this outcome was restricted to participants who reported having at least 1 child at Wave IV (*n* = 5,304).

ADHD, attention deficit hyperactivity disorder; CI, confidence interval; OR, odds ratio; PTSD, posttraumatic stress disorder; RR, risk ratio;.STI, sexually transmitted infection.

When considering psychological outcomes, positive affect was associated with healthier functioning, or scores, on 6 (out of 7) mental health outcomes, including: reduced likelihood of PTSD (odds ratio [*OR*] = 0·63, 95% CI = 0·46, 0·85, *p* = 0.003), reduced likelihood of ADD/ADHD (*OR* = 0·66, 95% CI = 0·45, 0·98, *p* = 0.039), reduced likelihood of anxiety diagnosis (*RR* = 0·81, 95% CI = 0·71, 0·93, *p* = 0.003), reduced likelihood of depression diagnosis (*RR* = 0·84, 95% CI = 0·74, 0·94, *p* = 0.004), lower perceived stress (β = −0·23, 95% CI = −0·30, −0·16, *p* < 0.001), and negative affect (β = −0·17, 95% CI = −0·24, −0·10, *p* < 0.001). However, there was little evidence of an association with suicidal ideation. Positive affect was also associated with healthier functioning, or scores, on 2 (out of 3) psychological well-being outcomes, including: optimism (β = 0·20, 95% CI = 0·12, 0·28, *p* < 0.001) and sense of control (β = 0·18, 95% CI = 0·13, 0·24, *p* < 0.001), but there was little evidence of association with job satisfaction.

When considering social outcomes, positive affect was associated healthier functioning, or scores, on 4 (out of 7) social factors, including: lower perceived discrimination (β = −0·10, 95% CI = −0·18, −0·02, *p* = 0.016) and loneliness (β = −0·09, 95% CI = −0·16, −0·02, *p* = 0.015), as well as higher satisfaction with parenting (β = 0·16, 95% CI = 0·08, 0·25, *p* < 0.001) and social support (β = 0·08, 95% CI = 0·02, 0·13, *p* = 0.008). However, there was little evidence of association with relationship quality with parent, social activities, or romantic relationship quality. Finally, positive affect was associated with 1 (out of 2) civic and prosocial behavior factors including: increased voting (*RR* = 1·25, 95% CI = 1·16, 1·36, *p* < 0.001). However, there was little evidence of association with volunteering.

We conducted 4 additional analyses. First, *E*-value analyses suggested that a few of the associations we observed were at least moderately robust to unmeasured confounding (Table 3). For example, an unmeasured confounder associated with both positive affect and anxiety diagnosis by risk ratios of 1·76, each, above, and beyond the large array of potential confounders already adjusted for, could explain away the association. However, weaker joint confounder associations could not. To shift the confidence interval to include the null, an unmeasured confounder associated with both positive affect and anxiety diagnosis by risk ratios of 1·36 each could suffice, but weaker joint confounder associations could not. However, several other associations were not especially robust to potential unmeasured confounding. Second, complete-case analyses provided similar results to those in the main analyses (Table C in S1 Appendix). Third, unadjusted models provided a baseline for comparison (Table D in S1 Appendix). Fourth, conventionally adjusted covariate models showed estimates that were stronger than the fully adjusted models (Table E in S1 Appendix). Fifth, we calculated the NNT and NNH for each outcome (see Table F in S1 Appendix).

**Table 3:** Robustness to unmeasured confounding (E-values) for the association between positive affect (3rd tertile vs. 1st tertile) in adolescence and subsequent health and well-being in adulthood (National Longitudinal Study of Adolescent to Adult Health [Add Health]).

Outcome	Effect estimate^a^	Confidence interval limit^b^
**Physical health**		
Number of diagnosed physical health conditions	1.23	1.00
Cancer	1.14	1.00
High cholesterol	1.42	1.00
Hypertension	1.48	1.00
Diabetes	1.58	1.00
Asthma	1.29	1.00
Sleep apnea	1.21	1.00
Migraines^c^	1.86	1.37
Allostatic load	1.29	1.00
Overweight/obesity	1.11	1.00
Functional limitations	1.68	1.00
Cognition^c^	1.47	1.26
Self-rated health	1.46	1.27
**Health behavior**		
Sleep disturbance	1.44	1.21
Physical inactivity	1.80	1.18
Cigarette smoking	1.35	1.00
Binge drinking	1.65	1.00
Marijuana use	1.06	1.00
Prescription drug misuse	2.13	1.36
Illicit drug use	1.41	1.00
History of STIs^c^	1.42	1.00
Preventative health care use^c^	1.23	1.00
**Mental health**		
Depression diagnosis	1.68	1.31
Anxiety diagnosis	1.76	1.36
PTSD diagnosis	2.58	1.64
ADD/ADHD diagnosis^c^	2.38	1.17
Negative affect	1.61	1.42
Suicidal ideation	2.06	1.00
Perceived stress	1.76	1.58
**Psychological well-being**		
Optimism	1.69	1.48
Job satisfaction^c^	1.31	1.00
Sense of control^c^	1.65	1.50
**Social factors**		
Relationship quality with parent	1.28	1.00
Social activities	1.31	1.00
Social support	1.35	1.16
Loneliness^c^	1.39	1.15
Romantic relationship quality^c^	1.28	1.00
Satisfaction with parenting^c^	1.59	1.37
Perceived discrimination	1.42	1.16
**Civic and prosocial behavior**		
Voting	1.82	1.58
Volunteering	1.32	1.00

The formula for calculating *E*-values can be found in VanderWeele and Ding (2017)

Outcomes were derived from Wave V unless otherwise noted.

^a^*E*-values for effect estimates are the minimum strength of association on the risk ratio scale that an unmeasured confounder would need to have with both the exposure and the outcome to fully explain away the observed association between the exposure and outcome, conditional on the measured covariates.

^b^*E*-values for the limit of the 95% CI closest to the null denote the minimum strength of association on the risk ratio scale that an unmeasured confounder would need to have with both the exposure and the outcome to shift the CI to include the null value, conditional on the measured covariates.

^c^Outcome was derived from data from Wave IV because the data for this outcome was not collected at Wave V.

ADHD, attention deficit hyperactivity disorder; CI, confidence interval; PTSD, posttraumatic stress disorder; STI, sexually transmitted infection.

## Discussion

In a nationally representative sample of U.S. adolescents, we observed that higher positive affect during adolescence was associated with many health/well-being outcomes in adulthood. These results were maintained after robust control for a wide range of potential confounders, as well as positive affect (and all the outcomes when available) in the prior wave. Positive affect was associated with most mental health outcomes (i.e., lower likelihood of PTSD diagnosis, ADD/ADHD diagnosis, anxiety diagnosis, and depression diagnosis, along with lower perceived stress and negative affect) and the majority of social outcomes (i.e., lower perceived discrimination and loneliness, as well as higher satisfaction with parenting and social support). It was also associated with some health behaviors (i.e., lower likelihood of prescription drug misuse, physical inactivity, and sleep disturbance) and a few physical health outcomes (i.e., lower likelihood of migraines, higher cognition, and self-rated health). Finally, positive affect was associated with most psychological well-being outcomes (i.e., higher optimism and sense of control), and also a civic/prosocial outcome (i.e., more voting). It is important to note that even by the end of follow-up, participants were relatively young and many physical health conditions typically emerge later in life.

Our results share some alignment with results from past work that evaluated “prevalence” of positive affect and outcomes. For example, consistent with past research which focused on adolescents and adults, we observed that “incident” positive affect was associated with some higher psychological well-being outcomes (e.g., sense of control and optimism) and better mental health outcomes (e.g., lower likelihood of/scores: negative affect, depression diagnosis, anxiety diagnosis, perceived stress) [33]. Also aligned with some past research we observed null associations (e.g., no associations with: smoking) [14]. However, our results also diverge with results from past research. For example, contrary to prior research which observed associations with some health behaviors [14,17,18], we did not observe associations with some health behaviors (e.g., not: smoking, binge drinking). We also did not observe associations with some physical health outcomes (e.g., healthier: BMI and composite biomarker scores) that past studies observed [14–16]. However, when considering our results that adjusted for only sociodemographic covariates we observed associations that align with prior research, such as associations with some health behaviors (i.e., lower likelihood of: smoking, binge drinking) and some health outcomes (i.e., composite biomarker score). Methodologically, the underlying reasons for diverging results between our study and past studies may stem from a range of sources including differences in: (1) which covariates were controlled for; (2) control for prior positive affect; (3) study population (e.g., nationally representative versus non-generalizable samples); (4) study design (e.g., cross-sectional versus longitudinal); (5) measurement of the exposure; and (6) measurement of the outcome (e.g., specific versus composite measures).

Our study has several limitations. First, both positive affect and most outcomes were self-reported; thus, potential self-report and common method bias is a concern. However, control for pre-baseline outcomes and a wide range of potential confounders helps to mitigate these concerns. Second, confounding by unmeasured variables and reverse causality are common concerns in observational research. However, controlling for a large array of variables, including the exposure and outcomes in the pre-baseline wave, the prospective nature of our data, and results from *E*-value analyses helps mitigate these concerns. Third, positive affect was derived from a scale originally designed to assess depressive symptoms. However, this subscale has demonstrated reliability and validity in various studies, and the positive affect dimension repeatedly emerges in factor analytics studies as illustrated by a meta-analysis [24], and has been used repeatedly in past research [25]. Future studies should consider using other positive affect scales (e.g., PANAS items, facial coding). Fourth, our study focused on U.S. adolescents, and its findings may not extend to other cultural contexts, where different cultural, social, and environmental factors can influence health and development, potentially affecting the applicability of our results internationally. Our study also has several important strengths including the use of a prospective, diverse, and nationally representative sample of adolescents. The study allowed us to evaluate evidence for a distinct question often of more interest to policy-makers and interventionists. However, only 1 year of positive affect exposure, which is what the design here evaluates, may be insufficiently long to substantially affect outcomes 10 to 12 or 19 to 22 years later. Evaluating the effects of longer positive affect exposure periods may be important.

An increasing number of countries are adopting well-being measures as critical tools for guiding policy choices [1]. Further, several countries seek innovative and cost-effective methods of enhancing the health/well-being of large adolescent populations. Evidence from both randomized controlled trials (aimed at individuals) [11] and case studies of successful policies (aimed at entire populations) [12] suggest positive affect can be enhanced. Our findings suggest that ongoing development and application of interventions and policies aimed at bolstering positive affect is a promising method of enhancing some aspects of health/well-being for our adolescent and emerging adult populations.

## Supporting information

S1 Appendix **Text A.** Assessment of Outcomes. **Text B.** Proof Illustrating How Controlling for Prior Levels of Positive Affect Can Help Us Evaluate How “Change” in Positive Affect is Associated with Subsequent Health and Well-Being Outcomes Over Time. **Table A.** Missing Data on Study Variables (National Longitudinal Study of Adolescent to Adult Health [Add Health]). **Table B.** Change in Positive Affect from the Pre-Baseline Wave (Wave I; t_0_) to the Baseline Wave (Wave II; t_1_). **Table C.** Associations of Positive Affect in Adolescence with Subsequent Health and Well-Being in Adulthood (Complete-Case Analyses; National Longitudinal Study of Adolescent to Adult Health [Add Health]). **Table D.** Associations of Positive Affect in Adolescence with Subsequent Health and Well-Being in Adulthood (Unadjusted or Fully Adjusted for Covariates; National Longitudinal Study of Adolescent to Adult Health [Add Health]). **Table E.** Associations of Positive Affect in Adolescence with Subsequent Health and Well-Being in Adulthood (Adjusting for Conventional Covariates or All Covariates; National Longitudinal Study of Adolescent to Adult Health [Add Health]). **Table F.** Associations of Positive Affect in Adolescence with Subsequent Health and Well-Being in Adulthood (Actual Amounts and/or Absolute Risks of Binary Outcomes; National Longitudinal Study of Adolescent to Adult Health [Add Health]). **Fig A.** Sample Inclusion Criteria for Positive Affect Analyses (Wave IV Outcomes). **Fig B.** Sample Inclusion Criteria for Positive Affect Analyses (Wave V Outcomes). **Checklist A.** Strengthening the Reporting of Observational Studies in Epidemiology (STROBE) Checklist.
(DOCX)

## Abbreviations

ADHD: attention deficit hyperactivity disorder
CES-D: Center for Epidemiological Studies Depression
CI: confidence interval
NNH: Number Needed to Harm
NNT: Number Needed to Treat
OR: odds ratio
PPVT: Peabody Picture Vocabulary Test
PTSD: posttraumatic stress disorder
RR: relative risk
STI: sexually transmitted infection
