# Supplementary material for: Positive affect during adolescence and health and well-being in adulthood: An outcome-wide longitudinal approach
Source: PLoS Med. 2024 Apr 2;21(4):e1004365. doi: 10.1371/journal.pmed.1004365 (PMC10986977; doi:10.1371/journal.pmed.1004365)
Supplement: S1 Appendix — Text A. Assessment of Outcomes. Text B. Proof Illustrating How Controlling for Prior Levels of Positive Affect Can Help Us Evaluate How “Change” in Positive Affect is Associated with Subsequent Health and Well-Being Outcomes Over Time. Table A. Missing Data on Study Variables (National Longitudinal Study of Adolescent to Adult Health [Add Health]). Table B. Change in Positive Affect from the Pre-Baseline Wave (Wave I; t0) to the Baseline Wave (Wave II; t1). Table C. Associations of Positive Affect in Adolescence with Subsequent Health and Well-Being in Adulthood (Complete-Case Analyses; National Longitudinal Study of Adolescent to Adult Health [Add Health]). Table D. Associations of Positive Affect in Adolescence with Subsequent Health and Well-Being in Adulthood (Unadjusted or Fully Adjusted for Covariates; National Longitudinal Study of Adolescent to Adult Health [Add Health]). Table E. Associations of Positive Affect in Adolescence with Subsequent Health and Well-Being in Adulthood (Adjusting for Conventional Covariates or All Covariates; National Longitudinal Study of Adolescent to Adult Health [Add Health]). Table F. Associations of Positive Affect in Adolescence with Subsequent Health and Well-Being in Adulthood (Actual Amounts and/or Absolute Risks of Binary Outcomes; National Longitudinal Study of Adolescent to Adult Health [Add Health]). Fig A. Sample Inclusion Criteria for Positive Affect Analyses (Wave IV Outcomes). Fig B. Sample Inclusion Criteria for Positive Affect Analyses (Wave V Outcomes). Checklist A. Strengthening the Reporting of Observational Studies in Epidemiology (STROBE) Checklist. (DOCX) [file pmed.1004365.s001.docx]

**Supplementary Online Content**

**S1 Appendix**

**Positive Affect During Adolescence and**

**Health and Well-Being in Adulthood: An Outcome-Wide Longitudinal Approach**

**Text A.** Assessment of Outcomes

**Text B.** Proof Illustrating How Controlling for Prior Levels of Positive Affect Can Help Us Evaluate How “Change” in Positive Affect is Associated with Subsequent Health and Well-Being Outcomes Over Time

**Table A.** Missing Data on Study Variables (National Longitudinal Study of Adolescent to Adult Health [Add Health])

**Table B.** Change in Positive Affect from the Pre-Baseline Wave (Wave I; t_0_) to the Baseline Wave (Wave II; t_1_)

**Table C.** Associations of Positive Affect in Adolescence with Subsequent Health and Well-Being in Adulthood (Complete-Case Analyses; National Longitudinal Study of Adolescent to Adult Health [Add Health])

**Table D.** Associations of Positive Affect in Adolescence with Subsequent Health and Well-Being in Adulthood (Unadjusted or Fully Adjusted for Covariates; National Longitudinal Study of Adolescent to Adult Health [Add Health])

**Table E.** Associations of Positive Affect in Adolescence with Subsequent Health and Well-Being in Adulthood (Adjusting for Conventional Covariates or All Covariates; National Longitudinal Study of Adolescent to Adult Health [Add Health])

**Table F.** Associations of Positive Affect in Adolescence with Subsequent Health and Well-Being in Adulthood (Actual Amounts and/or Absolute Risks of Binary Outcomes; National Longitudinal Study of Adolescent to Adult Health [Add Health])

**Fig A.** Sample Inclusion Criteria for Positive Affect Analyses (Wave IV Outcomes)

**Fig B.** Sample Inclusion Criteria for Positive Affect Analyses (Wave V Outcomes)

**Checklist A.** Strengthening the Reporting of Observational Studies in Epidemiology (STROBE) Checklist

**Text A**

**Assessment of Outcomes**

***Physical Health***

**Diagnosed Physical Health Conditions.** Participants self-reported (yes/no) whether they had ever been told by a doctor, nurse, or other health care provider that they had: a) cancer, b) high cholesterol, c) high blood pressure or hypertension (for women, when not pregnant), d) diabetes (for women, when not pregnant), e) asthma, f) migraine headaches (Wave IV only), or g) sleep apnea (Wave V only). Each of the conditions were summed, to create a composite score.

**Allostatic Load.** We created an allostatic load score for participants using biomarkers of stress, use of biomarker-regulating medications, and self-reports of health conditions related to the biomarkers. Add Health interviewers collected blood pressure, obtained blood samples for cholesterol and glucose from participants, and inventoried prescription medications during the in-home interviews. Following the approach used by Richardson, Goodwin, and Hummer (2021) [1], we determined the high-risk threshold for each biomarker and gave one point per biomarker value beyond that threshold. The high-risk threshold was above the 75^th^ percentile of all values in the sample for diastolic blood pressure (DBP), systolic blood pressure (SBP), pulse rate, waist circumference, hemoglobin A1c (HbA1c), and C-reactive protein (CRP); above the 80^th^ percentile for triglycerides and total cholesterol; and below the 20^th^ percentile for high-density lipoprotein (HDL) cholesterol. Among respondents who were not identified as high risk on a particular biomarker based on the sample-derived cutoffs, a point was given for use of a biomarker-regulating medication or a self-reported diagnosis of a condition related to a biomarker. In particular, for participants who were taking medication to manage hypertension, diabetes, hyperlipidemia, or inflammation, and/or those who reported receiving a diagnosis for hypertension, diabetes, or hyperlipidemia, a point was given for SBP, DBP, HbA1c, total cholesterol, triglycerides, HDL, or CRP, respectively. To calculate the total allostatic load score, the points based on biomarker levels, medication use, and self-reported conditions were summed, yielding a single score that ranged from 0 to 9 for each participant.

**Overweight/Obesity.** Add Health interviewers collected measures of participant weight and height and we calculated BMI (weight (lb) / [height (in)]^2^ x 703) and constructed categories of body mass index (BMI, kg/m^2^) as follows: underweight (<18.5), normal (18.5-<25), overweight (25-<30) or obese I (30-<35), obese II (35-<40), or obese III (40+). Participants with BMI categories of overweight or obese (I, II, or III) were assigned a code of 1 (*overweight/obese*) while those in the underweight or normal categories were coded as 0 (*not overweight/obese*).

**Functional Limitations.** Function limitations were assessed using an item asking participants about the extent to which their health limits their moderate activities (e.g., moving a table, pushing a vacuum cleaner, etc.). Responses indicating any limitations (i.e., “limited a little” or “limited a lot”) were coded as 1 (*functional limitations*) versus none (i.e., “not limited”) were coded as 0 (*no functional limitations*).

**Cognition.** Add Health interviewers performed three memory-ability tasks on participants: immediate word recall, delayed word recall, and digits backward recall. The word recall tasks involved interviewers reading a list of words and asking participants to relay back as many as they could remember in 90 seconds. The digit recall task used a set of numbers that participants were asked to recall back in reverse order, with interviewers repeating longer series of numbers each time up to seven times. The scores for each task were determined by the number of correct words [or numbers]. We standardized the scores on all three measures to have means of zero and standard deviations of one, and then summed the three measures together and re-standardized [2].

**Self-Rated Health.** Participants were asked “In general, how is your health?” with response categories including excellent, very good, good, fair, and poor. The measure was coded such that higher scores reflected better self-rated health.

***Health Behavior***

**Sleep Disturbance.** Sleep disturbance was assessed using two items asking participants over the past four weeks, “how often did you have trouble falling asleep?” and “how often did you have trouble staying asleep through the night?” Response options for both items included “never,” “less than once a week,” “1 or 2 times a week,” “3 or 4 times a week,” and “5 or more times a week.” We combined the two items for trouble falling and staying asleep to create a dichotomous indicator such that “had trouble falling (or staying) asleep 3 or 4 times a week or more in the past four weeks” was coded as 1 (*sleep disturbance*), and “had trouble falling (or staying) asleep 1 or 2 times a week or less” was coded as 0 (*no sleep disturbance)* [3].

**Physical Inactivity.** Participants were asked about their frequency of activity sessions across a range of different physical activities (e.g., bicycling, running, golfing, roller blading, walking for exercise) in the past seven days. Response options ranged from 0 (Not at all) to 7 (7 or more times), and the binary variable was created to by assigning 1 for no participation in the past week in any of the physical activities listed (*physical inactivity*) and 0 for any physical activity in the past week (*not physically inactive*).

**Cigarette Smoking.** Cigarette smoking was assessed by two items asking participants: 1) “Have you ever smoked cigarettes regularly--that is, at least one cigarette every day for 30 days?” and 2) During the past 30 days, on how many days did you smoke cigarettes?” Participants who reported they had ever smoked cigarettes regularly and smoked cigarettes on at least one of the past 30 days were coded 1 (*cigarette smoking*) and 0 for with never smoking regularly or not smoking at all in the past month (*no cigarette smoking*).

**Binge Drinking.** Participants were asked about their alcohol consumption during the past 12 months, including on how many days they drank 5 or more drinks in a row (if male; 4 or more if female). Response options were: ‘none,’ ‘1 or 2 days,’ ‘once a month or less (3 to 12 days in the past 12 months),’ ‘2 or 3 days a month,’ ‘1 or 2 days a week,’ ‘3 to 5 days a week,’ and ‘every day or almost every day.’ Those who reported binge drinking at least 1 or 2 days a week were assigned a code of 1 (*weekly binge drinking*), whereas those who reported binge drinking less than once a week or never were assigned a code of 0 (*no weekly binge drinking*) [4].

**Marijuana Use.** Marijuana use was assessed using an item that asked participants, “During the past 30 days, how many times did you use marijuana?” Responses options were: ‘never,’ ‘one day,’ ‘2 or 3 days,’ ‘1 day a week,’ ‘2 days a week,’ ‘3 to 5 days a week,’ and ‘every day or almost every day.’ Responses indicating any marijuana use in the past month were coded as 1 (*marijuana use*) and responses of ‘never’ were coded as 0 (*no marijuana use*).

**Prescription Drug Misuse.** Participants indicated whether they had in the past 30 days used any of the follow that “were not prescribed for you, taken in larger amounts than prescribed, more often than prescribed, for longer periods than prescribed, or that you took only for the feeling or experience they caused”: a) Sedatives or downers, such as barbiturates, sleeping pills, Quaalude, or Seconal; b) Tranquilizers, such as Librium, Valium, or Xanax; c) Stimulants or uppers, such as amphetamines, prescription diet pills, Ritalin, Preludin, or speed; Pain killers or opioids, such as Vicodin, OxyContin, Percocet, Demerol, Percodan, or Tylenol with codeine. Affirmative answers to taking any of the types of prescription drugs were coded as 1 (*prescription drug misuse)* and negative responses across all types of prescription drugs were coded as 0 (*no prescription drug misuse*).

**Illicit Drug Use.** Participants reported whether they had ever used any of the following drugs: a) steroids, b) cocaine, c) crystal meth, d) other. Any response of ‘yes’ was coded as 1 (*illicit drug use*) and responses of ‘no’ across all items were coded as 0 (*no illicit drug use*).

**History of Sexually Transmitted Infections.** Participants self-reported whether in the past 12 months they had been told by a doctor, nurse, or other health professional that they had any of the following sexually transmitted infections: a) chlamydia, b) gonorrhea, c) trichomoniasis, d) syphilis, e) genital herpes, f) genital warts, g) hepatitis B (HBV), h) human papilloma virus (HPV), i) pelvic inflammatory disease (PID), j) cervicitis or mucopurulent cervicitis (MPC), k) urethritis, l) vaginitis, m) HIV infection or AIDS, or n) any other sexually transmitted disease. Reports of any diagnosis of a sexually transmitted infections (STIs) were coded as 1 (*history of STIs*), while reports of no diagnoses across all types of sexually transmitted infections were coded as 0 (*no history of STIs*).

**Preventative Health Care Use.** Preventative health care use was assessed using an item that asked participants about how long ago they last had a routine check-up. Response options were ‘within the past 3 months,’ ‘4 to 6 months ago,’ ‘7 to 9 months ago,’ ‘10 to 12 months ago,’ ‘longer than 1 year ago but less than 2 years ago,’ ‘2 years ago or longer,’ or ‘never.’ Responses indicating the most recent health care visits were within the past 12 months were coded 1 (*preventative health care use*) and health care visits more than 12 months in the past were coded as 0 (*no preventative health care use*).

***Mental Health***

**Negative Affect***.* The measure for negative affect consists of three items that asked participants how often during the past seven days they experienced the following feelings: 1) blue, 2) depressed, and 3) sad. Response options were ‘never or rarely,’ ‘sometimes,’ ‘a lot of the time,’ and ‘most of the time or all of the time.’ The items were averaged together to create a composite score, with higher scores indicating higher depressive symptoms (α = 0.85; range: 1 to 4).

**Diagnosed Mental Health Conditions.** Participants self-reported whether they had ever been told by a doctor, nurse, or other health care provider that they have or had any of the following mental health conditions: a) depression, b) anxiety or panic disorder, c) post-traumatic stress disorder or PTSD, d) attention problems or ADD or ADHD. Each mental health condition was analyzed separately as a binary variable (no vs. yes).

**Suicidal Ideation.** Suicidal ideation was assessed by an item asking participants: “During the past 12 months, have you ever seriously thought about committing suicide?” Affirmative responses were coded 1 (*suicidal ideation*) and negative responses were coded 0 (*no suicidal ideation*).

**Perceived Stress.** Perceived stress was measured with four items asking participants how often in the last 30 days they felt: 1) they were unable to control the important things in their life, 2) they were confident in their ability to handle their personal problems, 3) things were going their way, and 4) difficulties were piling up so high that they could not overcome them. Response options included ‘never,’ ‘almost never,’ ‘sometimes,’ ‘fairly often,’ and ‘very often.’ After reverse coding positively worded items (items 2 and 3), the four items were averaged together to create a composite score, with higher scores indicating higher perceived stress (α = 0.78; range: 1 to 5) [5].

***Psychological Well-Being***

**Job Satisfaction.** Participants were asked about their satisfaction with their current or most recent job: “How satisfied (are/were) you with this job, as a whole?” Response categories ranged from 1 (extremely satisfied) to 5 (extremely dissatisfied), and the measure was reverse coded such that higher scores reflected greater job satisfaction.

**Optimism***.* Optimism was assessed using three items that asked participants about their level of agreement with statements about how they generally are now, not as they wish to be in the future: 1) “I'm always optimistic about my future,” 2) “I hardly ever expect things to go my way,” 3) “Overall, I expect more good things to happen to me than bad.” Response options ranged from 1 (strongly agree) to 5 (strongly disagree). After coding each item so that higher scores reflected stronger agreement with the positively valanced statements (items 1 and 3) and stronger disagreement with the negatively worded statement (items 2), the three items were averaged together to create a single composite measure with higher scores reflecting higher optimism (α = 0.68; range: 1 to 5) [6].

**Sense of Control***.* Sense of control was assessed using five items that asked participants about their level of agreement with the following statements: 1) “There is little I can do to change the important things in my life,” 2) “Other people determine most of what I can and cannot do,” 3) “There are many things that interfere with what I want to do,” 4) “There is really no way I can solve the problems I have,” and 5) “I am not interested in other people's problems.” Response options ranged from 1 (strongly agree) to 5 (strongly disagree). All items were reverse coded and then averaged together to create a composite measure, with higher scores indicating higher sense of control (α = 0.77, range:1 to 5) [7].

***Social Factors***

**Loneliness***.* Loneliness was assessed with an item that asked participants: “How often do you feel isolated from others?” Response options included ‘never,’ ‘rarely,’ ‘sometimes,’ and ‘often,’ with higher scores indicating higher loneliness.

**Romantic Relationship Quality.** We used seven items that asked participants about the extent to which they agreed or disagreed with the following statements about their relationship with their current or most recent partner: 1) “We [enjoy/enjoyed] doing even ordinary, day-to-day things together,” 2) “I [am/was] satisfied with the way we handle our problems and disagreements,” 3) “I [am/was] satisfied with the way we handle family finances,” 4) “My partner [listens/listened] to me when I need someone to talk to,” 5) “My partner [expresses/expressed] love and affection to me,” 6) “I [am/was] satisfied with our sex life,” and 7) “I [trust/trusted] my partner to be faithful to me.” Response options ranged from 1 (strongly agree) to 5 (strongly disagree). After reverse coding all items such that higher scores indicated stronger agreement with the statements, they were averaged together to create a composite measure, with higher scores indicating greater romantic relationship quality (α = 0.89, range: 1 to 5) [8].

**Parenting Satisfaction.** Parenting satisfaction was assessed using four items that asked participants who reported that they had children about their level of agreement with the following statements: 1) “I am happy in my role as a parent,” 2) “I feel close to my child(ren),” 3) “The major source of stress in my life is my child(ren),” and 4) “I feel overwhelmed by the responsibility of being a parent.” Response options ranged from 1 (strongly agree) to 5 (strongly disagree). After coding each item so that higher scores reflected stronger agreement with the positively valanced statements (items 1 and 2) and stronger disagreement with the negatively worded statements (items 3 and 4), the four items were averaged together to create a composite measure, with higher scores indicating higher parenting satisfaction (α = 0.58, range: 1 to 5) [9].

**Relationship Quality with a Parent.** Participants were asked about their relationships with their parental figures, including their level of closeness with their mother and/or father: “How close do you feel to your [mother/father figure]?” Response options included ‘not at all close,’ ‘not very close,’ ‘somewhat close,’ ‘quite close,’ and ‘very close.’ We used the maximum score between the two assessments (for closeness with mother and/or father), with higher scores indicating greater relationship quality with a parent.

**Social Activities.** Participants were asked: “In the past 12 months, how often did you get together socially with friends or relatives?” Response options were ‘never,’ ‘less than once a year,’ ‘about once or twice a year,’ ‘several times a year,’ ‘about once a month,’ ‘every week,’ and ‘several times a week.’ They were also asked how often in the past 12 months: “did you get together with any of your neighbors just to chat or for a social visit?” Responses for this item were ‘hardly ever,’ ‘several times a year,’ ‘several times a month,’ ‘several times a week,’ and ‘daily or almost every day.’ We created a binary measure for weekly participation, on average, in social activities with friends, relatives, or neighbors by coding responses of ‘every week’ or more frequently for the friends/relatives item or ‘several times a month,’ or more frequently for the neighbors item with 1 (*weekly social activities*) and responses of less frequently with 0 (*less than weekly social activities*).

**Social Support.** We created a measure for social support by using a series of items that asked about participants relationships with their spouse or partner, children, other family members, and friends. Participants indicated for each relationship type: “whether or not you can open up to them if you need to talk about your worries,” “whether or not you can rely on them for help if you have a problem,” and “whether or not they ever make too many demands or criticize you.” Response options were ‘yes’ or ‘no.’ Responses for the negatively valanced item (demands/criticism) were reverse coded, and then the items were summed to create a count measure for social support (α = 0.78, range: 0 to 12).

**Perceived Discrimination.** Participants were asked to indicate how often in their day-to-day life they experience any of the following: “You are treated with less courtesy or respect than other people,” “You receive poorer service than other people at restaurants or stores,” “People act as if they think you are not smart,” “People act as if they are afraid of you,” “You are threatened or harassed.” Response options included ‘never,’ ‘rarely,’ ‘sometimes,’ and ‘often.’ We used the average of the five items to create a composite perceived discrimination measure, with higher scores indicating more frequent experiences of perceived discrimination (α = 0.74, range: 1 to 4)

***Civic and Prosocial Behavior***

**Voting***.* Participants were asked about the following question about their voting behavior: “How often do you usually vote in local or statewide elections?” Response options included ‘never,’ ‘sometimes,’ ‘often,’ or ‘always.’ We created a binary measure with responses of voting ‘often’ or ‘always’ coded as 1 (*voting*) and responses of ‘sometimes’ or ‘never’ coded as 0 (*no voting*).

**Volunteering***.* Volunteering was assessed using an item that asked participants, “In the past 12 months, how many hours did you spend on volunteer or community service work?” Response categories included ‘0 hours,’ ‘1 to 19 hours,’ ‘20 to 39 hours,’ ‘40 to 79 hours,’ ‘80 to 159 hours,’ and ‘160 hours or more.’ We created a binary measure to represent volunteering in the past year by coding participants reporting any hours (i.e., 1 hour or more) of volunteering or community service work as 1 (*any volunteering*) and those reporting no hours of volunteering as 0 (*no volunteering*).

**Text B**

**Proof Illustrating How Controlling for Prior Levels of Positive Affect Can Help Us Evaluate How “Change” in Positive Affect is Associated with Subsequent Health and Well-Being Outcomes Over Time**

Let Y be the outcome in Wave IV, A_1_ the positive affect exposure in Wave II, A_0_ the positive affect exposure in Wave I, C the set of covariates in Wave I. For a continuous outcome, the regression model is: E[Y|a_0_, a_1_, c] = v + b_0_a_0_ b_1_a_1_+ b_2_’c

Let Y_a_ denote the potential outcome for Y for an individual under an intervention to set A_1_ to a. For an individual with baseline positive affect exposure A_0_=a_0_ and covariates c in Wave I, under the no-confounding (and positivity and consistency) and modeling assumptions, a change in positive affect of d points A_0_=a_0_ to A_1_=a_0_+d in Wave II, rather than maintaining positive affect of A_1_=a_0_ in Wave II, will give rise to an effect (a difference in potential outcomes for Y) of:

E[Y_a0+d_| A_0_=a_0_, c] - E[Y_a0_| A_0_=a_0_, c]

= E[Y_a0+d_| A_1_=a_0_+d, A_0_=a_0_, c] - E[Y_a0_| A_1_=a_0_, A_0_=a_0_, c]

= E[Y| A_1_=a_0_+d, A_0_=a_0_, c] - E[Y| A_1_=a_0_, A_0_=a_0_, c]

= [v + b_0_a_0_ + b_1_(a_0_+d) + b_2_’c] - [v + b_0_a_0_ + b_1_a_0_ + b_2_’c]

= b_1_d

where the first equality follows by the no-confounding assumption, the second by consistency, and the third by the statistical model.

**Note:** To evaluate potential “change” in positive affect we adjust for positive affect in the pre-baseline wave (t_0_). This analytic choice helps “hold constant” pre-baseline levels of positive affect. Therefore, those who have the highest levels of positive affect in the pre-baseline wave (t_0_) and continue having the highest levels of positive affect in the baseline wave (t_1_) contribute to the final estimate. However, the estimates produced from this analysis also corresponds to those who started in the lowest levels of positive affect in the pre-baseline wave (t_0_) and then moved to the middle or highest levels of positive affect in the baseline wave (t_1_), as well as all combinations of change in positive affect over time. Thus, readers are able to evaluate how change in positive affect (between t_0_ and t_1_), are associated with subsequent health and well-being outcomes.

By adjusting for prior values of the exposure, covariates, and outcomes it allowed us to evaluate “incident exposure” rather than “prevalent exposure.” This emphasis on incidence offers evidence for a distinct question, which is often of more interest to policy-makers and interventionists. What health and well-being outcomes might we observe if positive affect were intervened upon?

| **Table A**  **Missing Data on Study Variables (National Longitudinal Study of Adolescent to Adult Health [Add Health])** | | |
| --- | --- | --- |
|  | Missing | |
| Measure | *n* | %^a^ |
| Wave I covariates |  |  |
| Age | 17 | 0.08% |
| Female | 2 | 0.01% |
| Race/ethnicity | 21 | 0.10% |
| Born in the U.S. | 12 | 0.06% |
| Geographic region | 0 | 0.00% |
| Two-parent household | 25 | 0.12% |
| Number of siblings | 0 | 0.00% |
| Household income^b^ | 5394 | 26.00% |
| Household welfare receipt^b^ | 3407 | 16.42% |
| Has health insurance^b^ | 3062 | 14.76% |
| Smoker in household^b^ | 3158 | 15.22% |
| Mother age^b^ | 5070 | 24.44% |
| Mother race/ethnicity^b^ | 5133 | 24.74% |
| Parents born in the U.S. | 1040 | 5.01% |
| Parental education | 204 | 0.98% |
| Mother employed full-time | 170 | 0.82% |
| Mother religious service attendance^b^ | 5014 | 24.17% |
| Mother self-rated health^b^ | 4993 | 24.07% |
| Mother happy^b^ | 3206 | 15.45% |
| Parent has a disability | 1208 | 5.82% |
| Parent has obesity^b^ | 4083 | 19.68% |
| Parent has alcoholism^b^ | 4083 | 19.68% |
| Childhood maltreatment by parents^c^ | 208 | 1.00% |
| Mental health condition diagnosis^c^ | 0 | 0.00% |
| Negative affect | 72 | 0.35% |
| Self-esteem | 63 | 0.30% |
| Life expectancy | 137 | 0.66% |
| Parental control | 448 | 2.16% |
| Neighborhood social cohesion | 105 | 0.51% |
| Relationship quality with a parent | 452 | 2.18% |
| Religious service attendance | 35 | 0.17% |
| Has romantic partner | 256 | 1.23% |
| Has a learning disability^b^ | 3266 | 15.74% |
| PPVT | 1032 | 4.97% |
| School connectedness | 432 | 2.08% |
| GPA | 712 | 3.43% |
| Delinquency | 149 | 0.72% |
| Somatic symptoms | 23 | 0.11% |
| Pubertal development | 247 | 1.19% |
| Physical health condition diagnosis^c^ | 1465 | 9.33% |
| Overweight/obesity | 570 | 2.75% |
| Functional limitations | 259 | 1.25% |
| Self-rated health | 26 | 0.13% |
| Suicidal ideation | 244 | 1.18% |
| Sleep disturbance | 29 | 0.14% |
| Physical inactivity | 23 | 0.11% |
| Cigarette smoking | 144 | 0.69% |
| Binge drinking | 53 | 0.26% |
| Marijuana use | 216 | 1.04% |
| Illicit drug use | 416 | 2.01% |
| History of STIs | 40 | 0.19% |
| Preventative health care use | 73 | 0.35% |
| Positive affect | 42 | 0.20% |
| Wave II exposure |  |  |
| Positive affect | 11 | 0.07% |
| Wave IV outcomes |  |  |
| Migraines | 2 | 0.01% |
| History of STIs | 28 | 0.18% |
| Preventative health care use | 32 | 0.20% |
| ADD/ADHD diagnosis | 2 | 0.01% |
| Job satisfaction | 258 | 1.64% |
| Sense of control | 24 | 0.15% |
| Loneliness | 8 | 0.05% |
| Romantic relationship quality | 524 | 3.34% |
| Satisfaction with parenting^d^ | 25 | 0.32% |
| Wave V outcomes |  |  |
| Cancer | 60 | 0.49% |
| High cholesterol | 78 | 0.63% |
| Hypertension | 67 | 0.54% |
| Diabetes | 54 | 0.44% |
| Asthma | 60 | 0.49% |
| Sleep apnea | 45 | 0.37% |
| Allostatic load^e^ | 779 | 9.89% |
| Overweight/obesity | 83 | 0.67% |
| Functional limitations | 35 | 0.28% |
| Cognition | 62 | 0.50% |
| Self-rated health | 25 | 0.20% |
| Sleep disturbance | 19 | 0.15% |
| Physical inactivity | 364 | 2.96% |
| Cigarette smoking | 39 | 0.32% |
| Binge drinking | 21 | 0.17% |
| Marijuana use | 14 | 0.11% |
| Prescription drug misuse | 115 | 0.93% |
| Illicit drug use | 128 | 1.04% |
| Depression diagnosis | 55 | 0.45% |
| Anxiety diagnosis | 55 | 0.45% |
| PTSD diagnosis | 63 | 0.51% |
| Negative affect | 24 | 0.20% |
| Suicidal ideation | 277 | 2.25% |
| Perceived stress | 251 | 2.04% |
| Optimism | 215 | 1.75% |
| Relationship quality with parent | 1049 | 8.53% |
| Social activities | 243 | 1.98% |
| Social support | 287 | 2.33% |
| Perceived discrimination | 255 | 2.07% |
| Voting | 367 | 2.98% |
| Volunteering | 357 | 2.90% |
| ^a^Percent missing out of total responses at a given wave prior to sample restrictions: Wave I *N* = 20,745; Wave II *N* = 14,738; Wave IV *N* = 15,701; Wave V *N* = 12,300. | | |
| ^b^Derived from Wave I parent questionnaire, to which 3,075 adolescents did not have a parent respond (which are included in the *n* and % missing). | | |
| ^c^Retrospective item using Wave IV data so % missing corresponds to the Wave IV sample. | | |
| ^d^These items applied only to respondents who reported having children (*n* = 7,877) and the missingness information correspondents to respondents who were eligible for the items used to create the measure. | | |

| ^e^A subset of approximately 5,381 Wave V respondents provided biological samples that were used to construct the allostatic load measure and the missingness information corresponds to the missingness among those respondents who provided biological samples. | | | | |
| --- | --- | --- | --- | --- |
| **Table B**  **Change in Positive Affect from the Pre-Baseline Wave (Wave I; t_0_) to the Baseline Wave (Wave II; t_1_)^a,b,c^** | | | |  |
| **Positive affect** | **Baseline Wave (Wave II; t_1_)** | | |  |
|  | **Tertile 1** | **Tertile 2** | **Tertile 3** |  |
| **Pre-Baseline Wave (Wave I; t_0_)** | **%** | **%** | **%** |  |
| Tertile 1 | 2,644 (59·00) | 1,435 (32·02) | 402 (8·97) |  |
| Tertile 2 | 966 (32·78) | 1,379 (46·79) | 602 (20·43) |  |
| Tertile 3 | 567 (15·77) | 1,457 (40·53) | 1,571 (43·70) |  |
| ^a^The statistics presented are unweighted and sample is restricted to participants surveyed at the exposure wave (Wave II) and first outcome wave (Wave IV), and who had complete data on the exposure (*N* = 11,033).  ^b^The percentage of people in tertile 1, 2, or 3 in the pre-baseline wave (Wave I; t_0_) who one year later end up in tertile 1, 2, or 3 in the baseline wave (Wave II; t_1_). | | | | |
| ^c^The values in the first and third rows (tertiles 1 and 3) do not sum to 100% because of rounding. | | | | |

| **Table C**  **Associations of Positive Affect in Adolescence with Subsequent Health and Well-Being in Adulthood (Complete-Case Analyses; National Longitudinal Study of Adolescent to Adult Health [Add Health])** | | | | | | | |
| --- | --- | --- | --- | --- | --- | --- | --- |
|  | Positive affect | | | | | | |
|  | Tertile 1 | Tertile 2 | | | Tertile 3 | | |
| Outcome | (Reference) | β [95% CI] | RR/OR [95% CI] | *p*-value | β [95% CI] | RR/OR [95% CI] | *p*-value |
| **Physical health** |  |  |  |  |  |  |  |
| Number of diagnosed physical health conditions | 0.00 | 0.03 [-0.08, 0.15] | - | 0.545 | -0.03 [-0.17, 0.11] | - | 0.695 |
| Cancer | 1.00 | - | 1.18 [0.67, 2.09] | 0.568 | - | 0.70 [0.30, 1.61] | 0.397 |
| High cholesterol | 1.00 | - | 1.18 [0.86, 1.63] | 0.303 | - | 0.92 [0.64, 1.32] | 0.661 |
| Hypertension | 1.00 | - | 0.95 [0.79, 1.16] | 0.631 | - | 0.99 [0.79, 1.23] | 0.896 |
| Diabetes | 1.00 | - | 1.19 [0.75, 1.90] | 0.457 | - | 1.24 [0.62, 2.45] | 0.544 |
| Asthma | 1.00 | - | 1.00 [0.80, 1.24] | 0.973 | - | 0.95 [0.73, 1.25] | 0.716 |
| Sleep apnea | 1.00 | - | 0.96 [0.66, 1.41] | 0.846 | - | 0.81 [0.49, 1.35] | 0.421 |
| Migraines^a^ | 1.00 | - | 0.91 [0.75, 1.09] | 0.293 | - | 0.82 [0.66, 1.03] | 0.088 |
| Allostatic load | 0.00 | 0.02 [-0.10, 0.13] | - | 0.798 | 0.13 [-0.01, 0.28] | - | 0.066 |
| Overweight/obesity | 1.00 | - | 0.98 [0.87, 1.10] | 0.710 | - | 0.88 [0.76, 1.01] | 0.059 |
| Functional limitations | 1.00 | - | 1.08 [0.86, 1.34] | 0.512 | - | 0.77 [0.57, 1.04] | 0.084 |
| Cognition^a^ | 0.00 | 0.14 [0.04, 0.24] | - | 0.006 | 0.16 [0.04, 0.27] | - | 0.008 |
| Self-rated health | 0.00 | 0.02 [-0.07, 0.11] | - | 0.693 | 0.18 [0.09, 0.27] | - | <0.001 |
| **Health behavior** |  |  |  |  |  |  |  |
| Sleep disturbance | 1.00 | - | 0.95 [0.87, 1.03] | 0.194 | - | 0.90 [0.80, 1.00] | 0.048 |
| Physical inactivity | 1.00 | - | 0.92 [0.73, 1.16] | 0.459 | - | 0.92 [0.66, 1.28] | 0.613 |
| Cigarette smoking | 1.00 | - | 0.94 [0.82, 1.07] | 0.334 | - | 0.75 [0.62, 0.91] | 0.004 |
| Binge drinking | 1.00 | - | 0.83 [0.64, 1.08] | 0.159 | - | 0.68 [0.48, 0.96] | 0.027 |
| Marijuana use | 1.00 | - | 1.01 [0.85, 1.20] | 0.925 | - | 0.80 [0.62, 1.04] | 0.090 |
| Prescription drug misuse | 1.00 | - | 0.86 [0.66, 1.13] | 0.287 | - | 0.61 [0.41, 0.90] | 0.014 |
| Illicit drug use | 1.00 | - | 0.67 [0.47, 0.97] | 0.032 | - | 0.50 [0.29, 0.87] | 0.014 |
| History of STIs^a^ | 1.00 | - | 1.13 [0.86, 1.48] | 0.373 | - | 1.18 [0.89, 1.58] | 0.244 |
| Preventative health care use^a^ | 1.00 | - | 0.96 [0.90, 1.03] | 0.276 | - | 0.95 [0.88, 1.04] | 0.265 |
| **Mental health** |  |  |  |  |  |  |  |
| Depression diagnosis | 1.00 | - | 0.97 [0.84, 1.11] | 0.638 | - | 0.75 [0.64, 0.90] | 0.002 |
| Anxiety diagnosis | 1.00 | - | 0.89 [0.75, 1.05] | 0.176 | - | 0.73 [0.59, 0.89] | 0.003 |
| PTSD diagnosis | 1.00 | - | 0.76 [0.50, 1.16] | 0.204 | - | 0.64 [0.41, 1.00] | 0.052 |
| ADD/ADHD diagnosis^a^ | 1.00 | - | 1.07 [0.60, 1.90] | 0.819 | - | 0.93 [0.47, 1.82] | 0.827 |
| Negative affect | 0.00 | -0.08 [-0.17, 0.01] | - | 0.089 | -0.23 [-0.34, -0.12] | - | <0.001 |
| Suicidal ideation | 1.00 | - | 0.80 [0.54, 1.21] | 0.290 | - | 0.39 [0.23, 0.67] | <0.001 |
| Perceived stress | 0.00 | -0.12 [-0.21, -0.02] | - | 0.016 | -0.25 [-0.37, -0.12] | - | <0.001 |
| **Psychological well-being** |  |  |  |  |  |  |  |
| Optimism | 0.00 | 0.07 [-0.03, 0.17] | - | 0.166 | 0.23 [0.12, 0.34] | - | <0.001 |
| Job satisfaction^a^ | 0.00 | 0.08 [0.01, 0.16] | - | 0.034 | 0.04 [-0.06, 0.15] | - | 0.416 |
| Sense of control^a^ | 0.00 | 0.11 [0.03, 0.19] | - | 0.008 | 0.21 [0.11, 0.31] | - | <0.001 |
| **Social factors** |  |  |  |  |  |  |  |
| Relationship quality with parent | 0.00 | 0.06 [-0.02, 0.14] | - | 0.149 | 0.04 [-0.07, 0.15] | - | 0.449 |
| Social activities | 1.00 | - | 1.01 [0.92, 1.11] | 0.831 | - | 1.08 [0.99, 1.18] | 0.090 |
| Social support | 0.00 | 0.05 [-0.01, 0.11] | - | 0.093 | 0.08 [0.02, 0.14] | - | 0.014 |
| Loneliness^a^ | 0.00 | -0.03 [-0.11, 0.05] | - | 0.452 | -0.08 [-0.17, 0.01] | - | 0.082 |
| Romantic relationship quality^a^ | 0.00 | 0.08 [0.01, 0.16] | - | 0.034 | 0.06 [-0.04, 0.16] | - | 0.214 |
| Satisfaction with parenting^a,b^ | 0.00 | 0.14 [0.01, 0.26] | - | 0.028 | 0.18 [0.03, 0.32] | - | 0.016 |
| Perceived discrimination | 0.00 | -0.06 [-0.12, -0.01] | - | 0.031 | -0.07 [-0.13, -0.01] | - | 0.028 |
| **Civic and prosocial behavior** |  |  |  |  |  |  |  |
| Voting | 1.00 | - | 1.21 [1.10, 1.34] | <0.001 | - | 1.22 [1.08, 1.38] | 0.002 |
| Volunteering | 1.00 | - | 1.17 [1.03, 1.32] | 0.018 | - | 1.06 [0.92, 1.23] | 0.424 |
| *Note*. RR, risk ratio; OR, odds ratio; CI, confidence interval. | | |  |  |  |  |  |
| Outcomes were derived from Wave V and models were weighted by the Wave V sample weight unless otherwise noted. Outcomes associated with Wave V: *N* ranged from 4,116 to 4,375; outcomes associated with Wave IV: *N* ranged from 5,575 to 5,725. | | | | | | | |
| The analytic sample was restricted to those who participated in the survey at the exposure wave (Wave II) and had a valid sampling weight at the outcome wave from which the data for the respective outcome was derived (Wave IV or Wave V). Multiple imputation was performed to impute missing data on the covariates, exposure, and outcomes. All models controlled for sociodemographic and family factors (age, sex, race/ethnicity, nativity status, geographic region, family structure, number of siblings, household income, household welfare receipt, insurance status, smoker in household, mother age, mother race/ethnicity, parent nativity, parental education, mother employment status, mother religious service attendance, mother health status, mother happiness, parent has a disability, parent has obesity, parent has alcoholism, childhood maltreatment by parents), psychosocial and academic factors (mental health condition diagnosis, negative affect, self-esteem, life orientation, relationship quality with a parent, parental control, neighborhood social cohesion, religious service attendance, romantic relationship status, has a learning disability, PPVT, school connectedness, GPA, delinquency), health status and health behavior (somatic symptoms, pubertal development, physical health condition diagnosis, overweight/obesity, functional limitations, self-rated health, suicidal ideation, sleep disturbance, physical inactivity, cigarette smoking, binge drinking, marijuana use, illicit drug use, history of STIs, preventative health care use), and positive affect assessed at Wave I. | | | | | | | |
| An outcome-wide analytic approach was used, and a separate model was run for each outcome. A different type of model was run depending on the nature of the outcome: (1) for each binary outcome with a prevalence of ≥ 10%, a generalized linear model (with a log link and Poisson distribution) was used to estimate a RR; (2) for each binary outcome with a prevalence of < 10%, a logistic regression model was used to estimate an OR; and (3) for each continuous outcome, a linear regression model was used to estimate a β | | | | | | | |
| All continuous outcomes were standardized (mean = 0, standard deviation = 1), and β was the standardized effect size. | | | | | |  |  |
| ^a^Outcome was derived from data from Wave IV and model was weighted by the Wave IV sample weight because the data for this outcome was not collected at Wave V. | | | | | | | |
| ^b^Analysis for this outcome was restricted to participants who reported having at least one child at Wave IV (*n* = 2,586). | | | | | |  |  |

| **Table D**  **Associations of Positive Affect in Adolescence with Subsequent Health and Well-Being in Adulthood (Unadjusted or Fully Adjusted for Covariates; National Longitudinal Study of Adolescent to Adult Health [Add Health])** | | | | | | | |
| --- | --- | --- | --- | --- | --- | --- | --- |
|  | Positive affect | | | | | | |
|  |  | Unadjusted models^a^ | | | Fully-adjusted models^b^ | | |
|  | Tertile 1 | Tertile 3 | | | Tertile 3 | | |
| Outcome | (Reference) | β [95% CI] | RR/OR [95% CI] | *p*-value | β [95% CI] | RR/OR [95% CI] | *p*-value |
| **Physical health** |  |  |  |  |  |  |  |
| Number of diagnosed physical health conditions | 0.00 | -0.12 [-0.18, -0.06] | - | <0.001 | -0.04 [-0.11, 0.03] | - | 0.239 |
| Cancer | 1.00 | - | 0.86 [0.54, 1.38] | 0.526 | - | 1.02 [0.58, 1.77] | 0.957 |
| High cholesterol | 1.00 | - | 0.79 [0.65, 0.96] | 0.018 | - | 0.91 [0.72, 1.15] | 0.438 |
| Hypertension | 1.00 | - | 0.83 [0.73, 0.94] | 0.005 | - | 0.90 [0.79, 1.02] | 0.102 |
| Diabetes | 1.00 | - | 0.74 [0.56, 0.97] | 0.031 | - | 1.16 [0.79, 1.70] | 0.457 |
| Asthma | 1.00 | - | 0.94 [0.80, 1.10] | 0.426 | - | 0.95 [0.79, 1.14] | 0.558 |
| Sleep apnea | 1.00 | - | 0.89 [0.76, 1.05] | 0.170 | - | 0.97 [0.78, 1.21] | 0.767 |
| Migraines^c^ | 1.00 | - | 0.77 [0.66, 0.89] | <0.001 | - | 0.79 [0.67, 0.93] | 0.005 |
| Allostatic load | 0.00 | -0.15 [-0.24, -0.06] | - | 0.002 | -0.06 [-0.14, 0.02] | - | 0.149 |
| Overweight/obesity | 1.00 | - | 0.99 [0.92, 1.05] | 0.665 | - | 0.99 [0.91, 1.07] | 0.802 |
| Functional limitations | 1.00 | - | 0.61 [0.52, 0.71] | <0.001 | - | 0.84 [0.69, 1.01] | 0.066 |
| Cognition^c^ | 0.00 | 0.40 [0.34, 0.47] | - | <0.001 | 0.13 [-0.01, 0.27] | - | 0.002 |
| Self-rated health | 0.00 | 0.38 [0.31, 0.45] | - | <0.001 | 0.11 [0.05, 0.18] | - | <0.001 |
| **Health behavior** |  |  |  |  |  |  |  |
| Sleep disturbance | 1.00 | - | 0.86 [0.81, 0.91] | <0.001 | - | 0.91 [0.85, 0.97] | 0.004 |
| Physical inactivity | 1.00 | - | 0.63 [0.53, 0.76] | <0.001 | - | 0.80 [0.66, 0.98] | 0.029 |
| Cigarette smoking | 1.00 | - | 0.71 [0.63, 0.80] | <0.001 | - | 0.93 [0.83, 1.05] | 0.258 |
| Binge drinking | 1.00 | - | 0.89 [0.73, 1.08] | 0.226 | - | 0.85 [0.68, 1.05] | 0.123 |
| Marijuana use | 1.00 | - | 0.92 [0.80, 1.06] | 0.225 | - | 1.00 [0.87, 1.15] | 0.962 |
| Prescription drug misuse | 1.00 | - | 0.57 [0.46, 0.71] | <0.001 | - | 0.72 [0.56, 0.93] | 0.013 |
| Illicit drug use | 1.00 | - | 0.80 [0.55, 1.16] | 0.235 | - | 0.92 [0.61, 1.38] | 0.674 |
| History of STIs^c^ | 1.00 | - | 0.82 [0.68, 0.99] | 0.036 | - | 1.10 [0.92, 1.31] | 0.307 |
| Preventative health care use^c^ | 1.00 | - | 0.96 [0.92, 1.00] | 0.061 | - | 0.96 [0.92, 1.01] | 0.158 |
| **Mental health** |  |  |  |  |  |  |  |
| Depression diagnosis | 1.00 | - | 0.73 [0.65, 0.81] | <0.001 | - | 0.84 [0.74, 0.94] | 0.004 |
| Anxiety diagnosis | 1.00 | - | 0.75 [0.66, 0.84] | <0.001 | - | 0.81 [0.71, 0.93] | 0.003 |
| PTSD diagnosis | 1.00 | - | 0.52 [0.40, 0.67] | <0.001 | - | 0.63 [0.46, 0.85] | 0.003 |
| ADD/ADHD diagnosis^c^ | 1.00 | - | 0.68 [0.53, 0.86] | 0.002 | - | 0.66 [0.45, 0.98] | 0.039 |
| Negative affect | 0.00 | -0.36 [-0.43, -0.29] | - | <0.001 | -0.17 [-0.24, -0.10] | - | <0.001 |
| Suicidal ideation | 1.00 | - | 0.57 [0.43, 0.76] | <0.001 | - | 0.74 [0.52, 1.05] | 0.089 |
| Perceived stress | 0.00 | -0.44 [-0.50, -0.37] | - |  | -0.23 [-0.30, -0.16] | - | <0.001 |
| **Psychological well-being** |  |  |  |  |  |  |  |
| Optimism | 0.00 | 0.40 [0.33, 0.47] | - | <0.001 | 0.20 [0.12, 0.28] | - | <0.001 |
| Perceived discrimination | 0.00 | -0.26 [-0.34, -0.19] | - | <0.001 | -0.10 [-0.18, -0.02] | - | 0.081 |
| Job satisfaction^c^ | 0.00 | 0.15 [0.08, 0.21] | - | <0.001 | 0.06 [-0.01, 0.13] | - | <0.001 |
| Sense of control^c^ | 0.00 | 0.46 [0.41, 0.51] | - | <0.001 | 0.18 [0.13, 0.24] | - |  |
| **Social factors** |  |  |  |  |  |  | 0.279 |
| Relationship quality with parent | 0.00 | 0.11 [0.04, 0.18] | - | 0.002 | 0.04 [-0.03, 0.12] | - | 0.142 |
| Social activities | 1.00 | - | 1.18 [1.10, 1.25] | <0.001 | - | 1.06 [0.98, 1.14] | 0.008 |
| Social support | 0.00 | 0.18 [0.13, 0.23] | - | <0.001 | 0.08 [0.02, 0.13] | - | 0.015 |
| Loneliness^c^ | 0.00 | -0.20 [-0.27, -0.13] | - | <0.001 | -0.09 [-0.16, -0.02] | - | 0.121 |
| Romantic relationship quality^c^ | 0.00 | 0.18 [0.13, 0.24] | - | <0.001 | 0.05 [-0.01, 0.12] | - | <0.001 |
| Satisfaction with parenting^c,d^ | 0.00 | 0.34 [0.27, 0.41] | - | <0.001 | 0.16 [0.08, 0.25] | - | 0.016 |
| **Civic and prosocial behavior** |  |  |  |  |  |  |  |
| Voting | 1.00 | - | 1.52 [1.41, 1.64] | <0.001 | - | 1.25 [1.16, 1.36] | <0.001 |
| Volunteering | 1.00 | - | 1.36 [1.24, 1.50] | <0.001 | - | 1.06 [0.96, 1.18] | 0.260 |
| *Note*. RR, risk ratio; OR, odds ratio; CI, confidence interval. | | |  |  |  |  |  |
| Outcomes were derived from Wave V and models were weighted by the Wave V sample weight unless otherwise noted. Outcomes associated with Wave V: *N* = 9,003; outcomes associated with Wave IV: *N* = 11,040. | | | | | | | |
| The analytic sample was restricted to those who participated in the survey at the exposure wave (Wave II) and had a valid sampling weight at the outcome wave from which the data for the respective outcome was derived (Wave IV or Wave V). Multiple imputation was performed to impute missing data on the covariates, exposure, and outcomes. All models controlled for sociodemographic and family factors (age, sex, race/ethnicity, nativity status, geographic region, family structure, number of siblings, household income, household welfare receipt, insurance status, smoker in household, mother age, mother race/ethnicity, parent nativity, parental education, mother employment status, mother religious service attendance, mother health status, mother happiness, parent has a disability, parent has obesity, parent has alcoholism, childhood maltreatment by parents), psychosocial and academic factors (mental health condition diagnosis, negative affect, self-esteem, life orientation, relationship quality with a parent, parental control, neighborhood social cohesion, religious service attendance, romantic relationship status, has a learning disability, PPVT, school connectedness, GPA, delinquency), health status and health behavior (somatic symptoms, pubertal development, physical health condition diagnosis, overweight/obesity, functional limitations, self-rated health, suicidal ideation, sleep disturbance, physical inactivity, cigarette smoking, binge drinking, marijuana use, illicit drug use, history of STIs, preventative health care use), and positive affect assessed at Wave I. | | | | | | | |
| An outcome-wide analytic approach was used, and a separate model was run for each outcome. A different type of model was run depending on the nature of the outcome: (1) for each binary outcome with a prevalence of ≥ 10%, a generalized linear model (with a log link and Poisson distribution) was used to estimate a RR; (2) for each binary outcome with a prevalence of < 10%, a logistic regression model was used to estimate an OR; and (3) for each continuous outcome, a linear regression model was used to estimate a β. | | | | | | | |
| All continuous outcomes were standardized (mean = 0, standard deviation = 1), and β was the standardized effect size. | | | | | |  |  |
| ^a^All models include only positive affect assessed at Wave II. | | |  |  |  |  |  |
| ^b^All models controlled for sociodemographic and family factors (age, sex, race/ethnicity, nativity status, geographic region, family structure, number of siblings, household income, household welfare receipt, insurance status, smoker in household, mother age, mother race/ethnicity, parent nativity, parental education, mother employment status, mother religious service attendance, mother health status, mother happiness, parent has a disability, parent has obesity, parent has alcoholism, childhood maltreatment by parents), psychosocial and academic factors (mental health condition diagnosis, negative affect, self-esteem, life orientation, relationship quality with a parent, parental control, neighborhood social cohesion, religious service attendance, romantic relationship status, has a learning disability, PPVT, school connectedness, GPA, delinquency), health status and health behavior (somatic symptoms, pubertal development, physical health condition diagnosis, overweight/obesity, functional limitations, self-rated health, suicidal ideation, sleep disturbance, physical inactivity, cigarette smoking, binge drinking, marijuana use, illicit drug use, history of STIs, preventative health care use), and positive affect assessed at Wave I. | | | | | | | |
| ^c^Outcome was derived from data from Wave IV and model was weighted by the Wave IV sample weight because the data for this outcome was not collected at Wave V. | | | | | | | |
| ^d^Analysis for this outcome was restricted to participants who reported having at least one child at Wave IV (*n* = 5,304). | | | | | |  |  |

| **Table E**  **Associations of Positive Affect in Adolescence with Subsequent Health and Well-Being in Adulthood (Adjusting for Conventional Covariates or All Covariates; National Longitudinal Study of Adolescent to Adult Health [Add Health])** | | | | | | | |
| --- | --- | --- | --- | --- | --- | --- | --- |
|  | Positive affect | | | | | | |
|  |  | Conventionally-adjusted models^a^ | | | Fully-adjusted models^b^ | | |
|  | Tertile 1 | Tertile 3 | | | Tertile 3 | | |
| Outcome | (Reference) | β [95% CI] | RR/OR [95% CI] | *p*-value | β [95% CI] | RR/OR [95% CI] | *p*-value |
| **Physical health** |  |  |  |  |  |  |  |
| Number of diagnosed physical health conditions | 0.00 | -0.08 [-0.15, -0.02] | - | 0.008 | -0.04 [-0.11, 0.03] | - | 0.239 |
| Cancer | 1.00 | - | 0.91 [0.56, 1.48] | 0.712 | - | 1.02 [0.58, 1.77] | 0.957 |
| High cholesterol | 1.00 | - | 0.83 [0.67, 1.01] | 0.064 | - | 0.91 [0.72, 1.15] | 0.438 |
| Hypertension | 1.00 | - | 0.87 [0.76, 0.99] | 0.036 | - | 0.90 [0.79, 1.02] | 0.102 |
| Diabetes | 1.00 | - | 0.89 [0.67, 1.17] | 0.408 | - | 1.16 [0.79, 1.70] | 0.457 |
| Asthma | 1.00 | - | 0.97 [0.83, 1.13] | 0.697 | - | 0.95 [0.79, 1.14] | 0.558 |
| Sleep apnea | 1.00 | - | 0.91 [0.77, 1.09] | 0.296 | - | 0.97 [0.78, 1.21] | 0.767 |
| Migraines^c^ | 1.00 | - | 0.79 [0.69, 0.91] | 0.002 | - | 0.79 [0.67, 0.93] | 0.005 |
| Allostatic load | 0.00 | -0.12 [-0.21, -0.03] | - | 0.011 | -0.06 [-0.14, 0.02] | - | 0.149 |
| Overweight/obesity | 1.00 | - | 0.99 [0.93, 1.07] | 0.854 | - | 0.99 [0.91, 1.07] | 0.802 |
| Functional limitations | 1.00 | - | 0.68 [0.58, 0.79] | <0.001 | - | 0.84 [0.69, 1.01] | 0.066 |
| Cognition^c^ | 0.00 | 0.27 [0.21, 0.33] | - | 0.000 | 0.12 [0.05, 0.19] | - | 0.002 |
| Self-rated health | 0.00 | 0.30 [0.23, 0.37] | - | <0.001 | 0.11 [0.05, 0.18] | - | <0.001 |
| **Health behavior** |  |  |  |  |  |  |  |
| Sleep disturbance | 1.00 | - | 0.86 [0.81, 0.91] | <0.001 | - | 0.91 [0.85, 0.97] | 0.004 |
| Physical inactivity | 1.00 | - | 0.72 [0.61, 0.86] | <0.001 | - | 0.80 [0.66, 0.98] | 0.029 |
| Cigarette smoking | 1.00 | - | 0.74 [0.66, 0.82] | <0.001 | - | 0.93 [0.83, 1.05] | 0.258 |
| Binge drinking | 1.00 | - | 0.81 [0.67, 0.98] | 0.035 | - | 0.85 [0.68, 1.05] | 0.123 |
| Marijuana use | 1.00 | - | 0.87 [0.76, 1.00] | 0.043 | - | 1.00 [0.87, 1.15] | 0.962 |
| Prescription drug misuse | 1.00 | - | 0.61 [0.49, 0.76] | <0.001 | - | 0.72 [0.56, 0.93] | 0.013 |
| Illicit drug use | 1.00 | - | 0.81 [0.56, 1.16] | 0.245 | - | 0.92 [0.61, 1.38] | 0.674 |
| History of STIs^c^ | 1.00 | - | 0.87 [0.73, 1.04] | 0.117 | - | 1.10 [0.92, 1.31] | 0.307 |
| Preventative health care use^c^ | 1.00 | - | 0.99 [0.95, 1.04] | 0.767 | - | 0.96 [0.92, 1.01] | 0.158 |
| **Mental health** |  |  |  |  |  |  |  |
| Depression diagnosis | 1.00 | - | 0.72 [0.65, 0.81] | <0.001 | - | 0.84 [0.74, 0.94] | 0.004 |
| Anxiety diagnosis | 1.00 | - | 0.75 [0.67, 0.85] | <0.001 | - | 0.81 [0.71, 0.93] | 0.003 |
| PTSD diagnosis | 1.00 | - | 0.55 [0.42, 0.72] | <0.001 | - | 0.63 [0.46, 0.85] | 0.003 |
| ADD/ADHD diagnosis^c^ | 1.00 | - | 0.52 [0.41, 0.66] | <0.001 | - | 0.66 [0.45, 0.98] | 0.039 |
| Negative affect | 0.00 | -0.33 [-0.40, -0.27] | - | <0.001 | -0.17 [-0.24, -0.10] | - | <0.001 |
| Suicidal ideation | 1.00 | - | 0.55 [0.40, 0.74] | <0.001 | - | 0.74 [0.52, 1.05] | 0.089 |
| Perceived stress | 0.00 | -0.40 [-0.47, -0.33] | - | <0.001 | -0.23 [-0.30, -0.16] | - | <0.001 |
| **Psychological well-being** |  |  |  |  |  |  |  |
| Optimism | 0.00 | 0.38 [0.31, 0.45] | - | <0.001 | 0.20 [0.12, 0.28] | - | <0.001 |
| Job satisfaction^c^ | 0.00 | 0.13 [0.07, 0.20] | - | <0.001 | 0.06 [-0.01, 0.13] | - | 0.081 |
| Sense of control^c^ | 0.00 | 0.41 [0.36, 0.47] | - | <0.001 | 0.18 [0.13, 0.24] | - | <0.001 |
| **Social factors** |  |  |  |  |  |  |  |
| Relationship quality with parent | 0.00 | 0.09 [0.03, 0.16] | - | 0.008 | 0.04 [-0.03, 0.12] | - | 0.279 |
| Social activities | 1.00 | - | 1.12 [1.06, 1.20] | <0.001 | - | 1.06 [0.98, 1.14] | 0.142 |
| Social support | 0.00 | 0.16 [0.11, 0.21] | - | <0.001 | 0.08 [0.02, 0.13] | - | 0.008 |
| Loneliness^c^ | 0.00 | -0.21 [-0.28, -0.14] | - | <0.001 | -0.09 [-0.16, -0.02] | - | 0.015 |
| Romantic relationship quality^c^ | 0.00 | 0.15 [0.09, 0.21] | - | <0.001 | 0.05 [-0.01, 0.12] | - | 0.121 |
| Satisfaction with parenting^c,d^ | 0.00 | 0.32 [0.25, 0.39] | - | <0.001 | 0.16 [0.08, 0.25] | - | <0.001 |
| Perceived discrimination | 0.00 | -0.12 [-0.16, -0.08] | - | <0.001 | -0.10 [-0.18, -0.02] | - | 0.016 |
| **Civic and prosocial behavior** |  |  |  |  |  |  |  |
| Voting | 1.00 | - | 1.42 [1.32, 1.53] | <0.001 | - | 1.25 [1.16, 1.36] | <0.001 |
| Volunteering | 1.00 | - | 1.26 [1.14, 1.39] | <0.001 | - | 1.06 [0.96, 1.18] | 0.260 |
| *Note*. RR, risk ratio; OR, odds ratio; CI, confidence interval. | | |  |  |  |  |  |
| Outcomes were derived from Wave V and models were weighted by the Wave V sample weight unless otherwise noted. Outcomes associated with Wave V: *N* = 9,003; outcomes associated with Wave IV: *N* = 11,040. | | | | | | | |
| The analytic sample was restricted to those who participated in the survey at the exposure wave (Wave II) and had a valid sampling weight at the outcome wave from which the data for the respective outcome was derived (Wave IV or Wave V). Multiple imputation was performed to impute missing data on the covariates, exposure, and outcomes. All models controlled for sociodemographic and family factors (age, sex, race/ethnicity, nativity status, geographic region, family structure, number of siblings, household income, household welfare receipt, insurance status, smoker in household, mother age, mother race/ethnicity, parent nativity, parental education, mother employment status, mother religious service attendance, mother health status, mother happiness, parent has a disability, parent has obesity, parent has alcoholism, childhood maltreatment by parents), psychosocial and academic factors (mental health condition diagnosis, negative affect, self-esteem, life orientation, relationship quality with a parent, parental control, neighborhood social cohesion, religious service attendance, romantic relationship status, has a learning disability, PPVT, school connectedness, GPA, delinquency), health status and health behavior (somatic symptoms, pubertal development, physical health condition diagnosis, overweight/obesity, functional limitations, self-rated health, suicidal ideation, sleep disturbance, physical inactivity, cigarette smoking, binge drinking, marijuana use, illicit drug use, history of STIs, preventative health care use), and positive affect assessed at Wave I. | | | | | | | |
| An outcome-wide analytic approach was used, and a separate model was run for each outcome. A different type of model was run depending on the nature of the outcome: (1) for each binary outcome with a prevalence of ≥ 10%, a generalized linear model (with a log link and Poisson distribution) was used to estimate a RR; (2) for each binary outcome with a prevalence of < 10%, a logistic regression model was used to estimate an OR; and (3) for each continuous outcome, a linear regression model was used to estimate a β | | | | | | | |
| All continuous outcomes were standardized (mean = 0, standard deviation = 1), and β was the standardized effect size. | | | | | |  |  |
| ^a^All models controlled for age, sex, race/ethnicity, nativity status, geographic region, family structure, household income, and parental education assessed at Wave I. | | | | | | | |
| ^b^All models controlled for sociodemographic and family factors (age, sex, race/ethnicity, nativity status, geographic region, family structure, number of siblings, household income, household welfare receipt, insurance status, smoker in household, mother age, mother race/ethnicity, parent nativity, parental education, mother employment status, mother religious service attendance, mother health status, mother happiness, parent has a disability, parent has obesity, parent has alcoholism, childhood maltreatment by parents), psychosocial and academic factors (mental health condition diagnosis, negative affect, self-esteem, life orientation, relationship quality with a parent, parental control, neighborhood social cohesion, religious service attendance, romantic relationship status, has a learning disability, PPVT, school connectedness, GPA, delinquency), health status and health behavior (somatic symptoms, pubertal development, physical health condition diagnosis, overweight/obesity, functional limitations, self-rated health, suicidal ideation, sleep disturbance, physical inactivity, cigarette smoking, binge drinking, marijuana use, illicit drug use, history of STIs, preventative health care use), and positive affect assessed at Wave I. | | | | | | | |
| ^c^Outcome was derived from data from Wave IV and model was weighted by the Wave IV sample weight because the data for this outcome was not collected at Wave V. | | | | | | | |
| ^d^Analysis for this outcome was restricted to participants who reported having at least one child at Wave IV (*n* = 5,304). | | | | | |  |  |

| **Table F**  **Associations of Positive Affect in Adolescence with Subsequent Health and Well-Being in Adulthood (Actual Amounts and/or Absolute Risks of Binary Outcomes; National Longitudinal Study of Adolescent to Adult Health [Add Health])** | | | | | |
| --- | --- | --- | --- | --- | --- |
|  | Positive affect | | | | |
|  | Tertile 1 | | | | Tertile 3 |
| Outcome | Number of Cases  (*n*) | Prevalence (%) | ARR  (%) | NNT/NNH | RR/OR  [95% CI] |
| **Physical health** |  |  |  |  |  |
| Diagnosed physical health conditions |  |  |  |  |  |
| Cancer | N/A | N/A | N/A | N/A | N/A |
| High cholesterol | N/A | N/A | N/A | N/A | N/A |
| Hypertension | N/A | N/A | N/A | N/A | N/A |
| Diabetes | N/A | N/A | N/A | N/A | N/A |
| Asthma | N/A | N/A | N/A | N/A | N/A |
| Sleep apnea | N/A | N/A | N/A | N/A | N/A |
| Migraines^a^ | 654 | 5.92% | 1.24% | 81 | 0.79 [0.67, 0.93] |
| Overweight/obesity | N/A | N/A | N/A | N/A | N/A |
| Functional limitations^b^ | 917 | 10.19% | 1.63% | 68 | 0.84 [0.69, 1.01] |
| **Health behavior** |  |  |  |  |  |
| Sleep disturbance | 1940 | 21.55% | 1.94% | 52 | 0.91 [0.85, 0.97] |
| Physical inactivity | 491 | 5.45% | 1.09% | 92 | 0.80 [0.66, 0.98] |
| Cigarette smoking | N/A | N/A | N/A | N/A | N/A |
| Binge drinking | N/A | N/A | N/A | N/A | N/A |
| Marijuana use | N/A | N/A | N/A | N/A | N/A |
| Prescription drug misuse | 464 | 5.15% | 1.44% | 70 | 0.72 [0.56, 0.93] |
| Illicit drug use | N/A | N/A | N/A | N/A | N/A |
| History of STIs^a^ | N/A | N/A | N/A | N/A | N/A |
| Preventative health care use^a^ | N/A | N/A | N/A | N/A | N/A |
| **Mental health** |  |  |  |  |  |
| Depression diagnosis | 943 | 10.47% | 1.68% | 60 | 0.84 [0.74, 0.94] |
| Anxiety diagnosis | 869 | 9.65% | 1.83% | 55 | 0.81 [0.71, 0.93] |
| PTSD diagnosis^b^ | 266 | 2.95% | 1.09% | 94 | 0.63 [0.46, 0.85] |
| ADD/ADHD diagnosis^a,b^ | 233 | 2.59% | 0.88% | 116 | 0.66 [0.45, 0.98] |
| Suicidal ideation | N/A | N/A | N/A | N/A | N/A |
| **Social factors** |  |  |  |  |  |
| Social activities | N/A | N/A | N/A | N/A | N/A |
| **Civic and prosocial behavior** |  |  |  |  |  |
| Voting | 1277 | 14.18% | -3.55% | -28 | 1.25 [1.16, 1.36] |
| Volunteering | N/A | N/A | N/A | N/A | N/A |
| *Note*. ARR, absolute risk reduction; NNT, number needed to treat; NNH, number needed to harm; RR, risk ratio; OR, odds ratio; CI, confidence interval. | | | | | |
| ARR = Prevalence-(RR*Prevalence); NNT/NNH = 1/ARR. | | |  |  |  |
| Outcomes were derived from Wave V and models were weighted by the Wave V sample weight unless otherwise noted. Outcomes associated with Wave V: *N* = 9,003; outcomes associated with Wave IV: *N* = 11,040. | | | | | |
| ^a^Outcome was derived from data from Wave IV because the data for this outcome was not collected at Wave V.  ^b^To calculate NNT from Odds Ratios (OR), the following formula was applied: NNT = (1-(PEER*(1-OR)))/((1-PEER)*PEER*(1-OR)), where PEER represents the Patient's Expected Event Rate. | | | | | |

**Figure A. Sample Inclusion Criteria for Positive Affect Analyses (Wave IV Outcomes)**

Completed Wave I in-home survey: 20,745

Did not participate in Wave II survey: 6,009

Completed Wave II in-home survey: 14,736

Did not participate in Wave IV survey: 2,873

Completed Wave IV survey: 11,863

Non-valid Wave IV survey weight: 823

Final sample for Wave IV outcomes: 11,040

**Figure B. Sample Inclusion Criteria for Positive Affect Analyses (Wave V Outcomes)**

Completed Wave I in-home survey: 20,745

Did not participate in Wave II survey: 6,009

Completed Wave II in-home survey: 14,736

Did not participate in Wave V survey: 5,507

Completed Wave V survey: 9,229

Non-valid Wave V survey weight: 226

Final sample for Wave V outcomes: 9,003

**Checklist A.** Strengthening the Reporting of Observational Studies in Epidemiology (STROBE) Checklist

|  | Item No | Recommendation | Page No |
| --- | --- | --- | --- |
| **Title and abstract** | 1 | (*a*) Indicate the study’s design with a commonly used term in the title or the abstract | Title |
|  |  | (*b*) Provide in the abstract an informative and balanced summary of what was done and what was found | Abstract |
| Introduction | | | |
| Background/rationale | 2 | Explain the scientific background and rationale for the investigation being reported | Introduction, Paragraphs 1, 2 |
| Objectives | 3 | State specific objectives, including any prespecified hypotheses | Introduction, Paragraph 4 |
| Methods | | | |
| Study design | 4 | Present key elements of study design early in the paper | Methods, Paragraph 1, 2 |
| Setting | 5 | Describe the setting, locations, and relevant dates, including periods of recruitment, exposure, follow-up, and data collection | Methods, Paragraphs 1, 2 |
| Participants | 6 | (*a*) Give the eligibility criteria, and the sources and methods of selection of participants. Describe methods of follow-up | Methods, Paragraph 1 |
|  |  | (*b*) For matched studies, give matching criteria and number of exposed and unexposed | N/A |
| Variables | 7 | Clearly define all outcomes, exposures, predictors, potential confounders, and effect modifiers. Give diagnostic criteria, if applicable | Measures Paragraphs 1-3 |
| Data sources/ measurement | 8* | For each variable of interest, give sources of data and details of methods of assessment (measurement). Describe comparability of assessment methods if there is more than one group | Measures Paragraphs 1-3, Text A |
| Bias | 9 | Describe any efforts to address potential sources of bias | Statistical Analysis Paragraph 5 |
| Study size | 10 | Explain how the study size was arrived at | Methods Paragraph 1, Figures A and B in S1 Appendix |
| Quantitative variables | 11 | Explain how quantitative variables were handled in the analyses. If applicable, describe which groupings were chosen and why | Statistical Analysis Paragraphs 1-4 |
| Statistical methods | 12 | (*a*) Describe all statistical methods, including those used to control for confounding | Statistical Analysis Paragraphs 1-4 |
|  |  | (*b*) Describe any methods used to examine subgroups and interactions | N/A |
|  |  | (*c*) Explain how missing data were addressed | Statistical Analysis Paragraph 5 |
|  |  | (*d*) If applicable, explain how loss to follow-up was addressed | Statistical Analysis Paragraph 5 |
|  |  | (*e*) Describe any sensitivity analyses | Statistical Analysis Paragraph 4 |
| Results | | |  |
| Participants | 13* | (a) Report numbers of individuals at each stage of study—eg numbers potentially eligible, examined for eligibility, confirmed eligible, included in the study, completing follow-up, and analysed | Results, Paragraph 1 |
|  |  | (b) Give reasons for non-participation at each stage | Figs A, B in S1 Appendix |
|  |  | (c) Consider use of a flow diagram | Figs A, B in S1 Appendix |
| Descriptive data | 14* | (a) Give characteristics of study participants (eg demographic, clinical, social) and information on exposures and potential confounders | Results, Paragraph 1 |
|  |  | (b) Indicate number of participants with missing data for each variable of interest | Table A in S1 Appendix |
|  |  | (c) Summarise follow-up time (eg, average and total amount) | Results, Paragraph 1 |
| Outcome data | 15* | Report numbers of outcome events or summary measures over time | Table S5 |
| Main results | 16 | (*a*) Give unadjusted estimates and, if applicable, confounder-adjusted estimates and their precision (eg, 95% confidence interval). Make clear which confounders were adjusted for and why they were included | Results, Paragraph 2-4 |
|  |  | (*b*) Report category boundaries when continuous variables were categorized | N/A |
|  |  | (*c*) If relevant, consider translating estimates of relative risk into absolute risk for a meaningful time period | Table F in Appendix S1 |
| Other analyses | 17 | Report other analyses done—eg analyses of subgroups and interactions, and sensitivity analyses | Results, Paragraph 5 |
| Discussion | | | |
| Key results | 18 | Summarise key results with reference to study objectives | Discussion Paragraph 1 |
| Limitations | 19 | Discuss limitations of the study, taking into account sources of potential bias or imprecision. Discuss both direction and magnitude of any potential bias | Discussion Paragraph 3 |
| Interpretation | 20 | Give a cautious overall interpretation of results considering objectives, limitations, multiplicity of analyses, results from similar studies, and other relevant evidence | Discussion Paragraph 4 |
| Generalisability | 21 | Discuss the generalisability (external validity) of the study results | Discussion Paragraph 3 |
| Other information | | | |
| Funding | 22 | Give the source of funding and the role of the funders for the present study and, if applicable, for the original study on which the present article is based | Funding |

*Give information separately for exposed and unexposed groups.

**Note:** An Explanation and Elaboration article discusses each checklist item and gives methodological background and published examples of transparent reporting. The STROBE checklist is best used in conjunction with this article (freely available on the Web sites of PLoS Medicine at http://www.plosmedicine.org/, Annals of Internal Medicine at http://www.annals.org/, and Epidemiology at http://www.epidem.com/). Information on the STROBE Initiative is available at http://www.strobe-statement.org.

**References**

1. Richardson LJ, Goodwin AN, Hummer RA. Social status differences in allostatic load among young adults in the United States. *SSM Popul Health.* 2021;15:100771.
2. Stebbins RC, Yang YC, Reason M, Aiello AE, Belsky DW, Harris KM, et al. Occupational cognitive stimulation, socioeconomic status, and cognitive functioning in young adulthood. *SSM Popul Health.* 2022;17:101024.
3. Fricke J, Sironi M. Dimensions of sexual orientation and sleep disturbance among young adults. *Prev Med Rep.* 2017;8:18-24.
4. Patrick, ME, Schulenberg, JE. Prevalence and predictors of adolescent alcohol use and binge drinking in the United States. *Alcohol Res*. 2014; 35(2): 193–200.
5. Lippert AM, Damaske S. Finding jobs, forming families, and stressing out? Work, family, and stress among young adult women in the United States. *Soc Forces.* 2019;98(2):885-914.
6. Fletcher J. Assessing the importance of childhood context in the development of hope and optimism. *J Happiness Stud.* 2020;21:2419-2427.
7. McFarland MJ, Wagner B, Marklin S. College education and sense of control: A twin-discordant design. *Socius.* 2016;2:2378023116656011.
8. Washington C. Romantic relationship quality of youth with two biological parents and stepfathers. *J Fam Issues.* 2021;42(6):1333-1353.
9. Beaver KM, da Silva Costa C, Poersch AP, Freddi MC, Stelmach MC, Connolly EJ, et al. Psychopathic personality traits and their influence on parenting quality: Results from a nationally representative sample of Americans. *Psychiatr Q*. 2014;85:497-511.
